# Manufacturing and electrostatic surface optimization of CAR-T cells improve therapeutic efficacy in liver malignancies

**DOI:** 10.1097/HC9.0000000000000982

**Published:** 2026-07-15

**Authors:** Linke Bian, Jiufei Zhu, Hongye Wang, Zhaorong Wu, Zhigang Zheng, Yuan Peng, Ying Liu, Xu Fang, Xiaochun Huang, Tianyang Jie, Zheyu Zhang, Hao Zhang, Fan Yang, Dan Cui, Bo Zhai, Feng Xue

**Affiliations:** 1 Department of Liver Surgery, Renji Hospital, Shanghai Jiao Tong University School of Medicine, Shanghai, China; 2 Department of Biliary-Pancreatic Surgery, Renji Hospital, Shanghai Jiao Tong University School of Medicine, Shanghai, China; 3 Mini-invasive Interventional Therapy Center, Shanghai East Hospital, Tongji University, Shanghai, China; 4 School of Life Sciences, Xiamen University, Fujian, China; 5 Department of Orthopedics, Renji Hospital, Shanghai Jiao Tong University School of Medicine, Shanghai, China; 6 Department of Pharmacy, Renji Hospital, Shanghai Jiao Tong University, Shanghai, China

**Keywords:** adoptive cell therapy, positively charged patches (PCPs), protein aggregation, syngeneic model, tonic signaling

## Abstract

**Background:**

Chimeric antigen receptor T-cell (CAR-T) therapy has achieved remarkable success in hematologic malignancies, yet demonstrates limited efficacy in solid tumors, including hepatic cancers. T-cell exhaustion and insufficient persistence represent major obstacles. We hypothesized that optimizing both manufacturing processes and CAR structural design could reduce exhaustion and enhance therapeutic outcomes in immunocompetent mouse models of primary and metastatic liver malignancies.

**Methods:**

We systematically compared antibody-based versus bead-based activation methods, evaluating their effects on T-cell exhaustion phenotypes. Retroviral vector (RVV) production was optimized for murine T-cell transduction, assessing vector stability and T-cell phenotypes. Different cytokine conditions were tested for their impact on T-cell expansion, memory phenotypes, and anti-tumor efficacy using GPC3-targeted CAR-T cells in hepatocellular carcinoma models. Through structural prediction and electrostatic field simulation, we identified that high positive charge patches (PCP) in the EpCAM-targeting G8.8 scFv caused CAR clustering and tonic signaling. We generated charge-optimized variants and evaluated their therapeutic efficacy in an immunocompetent colorectal cancer liver metastasis model.

**Results:**

Antibody activation showed superior homogeneity and expansion despite initially higher exhaustion markers, which equilibrated by day 10 without affecting viability. RVV harvested at 72 hours post-transfection yielded optimal titers. RVV remained stable through freeze–thaw cycles. IL-7 supplementation to IL-2 significantly enhanced memory phenotypes, reduced exhaustion, and improved tumor control in GPC3-CAR-T therapy. Electrostatic optimization of G8.8 scFv substantially reduced tonic signaling, decreased T-cell exhaustion, and enhanced anti-tumor efficacy in the MC38-EpCAM model.

**Conclusions:**

Systematic optimization of manufacturing conditions and structure-based charge engineering of CAR constructs synergistically enhance therapeutic efficacy against liver malignancies, providing a translatable framework for improving solid tumor CAR-T therapy.

## INTRODUCTION

Chimeric antigen receptor (CAR) T-cell therapy has revolutionized the treatment of hematologic malignancies, with remarkable clinical success in B-cell leukemias and lymphomas.^1^ However, the translation of this therapeutic modality to solid tumors has met with limited success.^2^ Key obstacles include insufficient CAR-T cell trafficking to tumors, limited expansion and persistence, and T-cell exhaustion induced by the hostile tumor microenvironment (TME).^3^ The immunosuppressive TME of solid tumors fundamentally differs from the milieu encountered in hematologic malignancies, contributing to premature CAR-T cell dysfunction and therapeutic failure.^4^

T-cell exhaustion represents a major barrier to effective CAR-T therapy in solid tumors.^5^ Exhausted T cells exhibit progressive loss of effector functions, sustained expression of inhibitory receptors such as PD-1, TIM-3, and LAG-3, and impaired proliferative capacity.^6^ In CAR-T cells, exhaustion can arise from multiple sources, including chronic antigen stimulation, immunosuppressive signals within the TME, and, importantly, antigen-independent tonic signaling through the CAR itself.^7^ Tonic signaling, caused by spontaneous CAR clustering and activation in the absence of target antigen, drives premature differentiation and exhaustion, severely compromising therapeutic efficacy.^8^ Recent studies have demonstrated that electrostatic interactions, particularly positive charge patches (PCPs) on the CAR surface, promote CAR clustering and tonic signaling.^9^ Tuning the charge density of CAR molecules through rational engineering of the single-chain variable fragment (scFv) has emerged as a promising strategy to optimize tonic signaling and enhance CAR-T cell fitness.^9^

Beyond structural optimization, manufacturing processes profoundly influence CAR-T cell quality, particularly memory phenotype and exhaustion status.^10^ T-cell activation represents the initial critical step in CAR-T manufacturing.^11^^,^^12^ Importantly, the strength and duration of initial activation signals can predetermine T-cell fate, influencing the balance between effector and memory differentiation.^13^

The cytokine milieu during ex vivo expansion critically shapes CAR-T cell phenotype and functional properties.^14^ Conventional manufacturing protocols rely heavily on interleukin-2 (IL-2), which promotes robust T-cell expansion but drives terminal effector differentiation and limits memory formation.^15^ Homeostatic cytokines such as IL-7 and IL-15 support memory T-cell development and long-term persistence.^16^ IL-7 and IL-15 preserve T memory stem cells (T_SCM_) and central memory T cells (T_CM_), populations associated with superior in vivo expansion and anti-tumor efficacy.^17^ Optimizing cytokine conditions to balance expansion with memory preservation represents a key manufacturing parameter warranting systematic investigation.

Efficient genetic modification of murine T cells poses distinct technical challenges compared with human T cells.^18^ Retroviral transduction of primary mouse T cells typically yields lower efficiency than human counterparts, necessitating optimization of vector production, transduction conditions, and the use of enhancing reagents.^19^ Polybrene and retronectin, cationic polymers and recombinant fibronectin fragments, respectively, facilitate viral vector binding to target cells and enhance transduction efficiency.^20^ However, their optimal concentrations and potential impact on T-cell viability and phenotype require careful titration. Furthermore, retroviral vector (RVV) stability during production, storage, and freeze–thaw cycles directly impacts manufacturing reproducibility and scalability.^21^

Immunocompetent syngeneic mouse models offer critical advantages over immunodeficient xenograft systems for preclinical CAR-T evaluation.^22^ Syngeneic models enable investigation of CAR-T cell interactions with an intact host immune system, providing more physiologically relevant assessment of therapeutic efficacy, toxicity, and resistance mechanisms.^23^ Murine CAR-T cells in immunocompetent mice recapitulate key aspects of human CAR-T therapy, including cytokine release, immune cell crosstalk, and TME remodeling that cannot be adequately modeled in immunodeficient hosts.^24^

The present study systematically addresses these gaps through integrated optimization of manufacturing and structure-based CAR engineering in immunocompetent mouse models. We compared activation methods, optimized retroviral transduction protocols, evaluated cytokine conditions, and applied electrostatic surface optimization to reduce tonic signaling and exhaustion. Our findings provide a translatable framework for enhancing CAR-T efficacy against primary and metastatic liver cancers.

## METHODS

### Cell culture

Murine hepatocellular carcinoma cell line Hepa1-6 engineered to stably express chimeric (human-murine hybrid) GPC3 (Hepa1-6-chGPC3, which was provided by CARsgen Therapeutics Co., Ltd.), murine colorectal carcinoma cell line MC38 stably transduced to express human EpCAM (MC38-EpCAM), and retroviral packaging cell lines Phoenix-Eco and PlatE-Eco were maintained in complete DMEM-GlutaMAX medium (Gibco, Thermo Fisher Scientific). Tumor cell lines were authenticated by short tandem repeat profiling and routinely tested for mycoplasma contamination. Sorted primary murine T cells were cultured in RPMI 1640 ATCC modification medium (Gibco) containing 2 mM L-glutamine, 10 mM HEPES, 1 mM sodium pyruvate, 4500 mg/L glucose, and 1500 mg/L sodium bicarbonate, further supplemented with 10% heat-inactivated FBS, 100 U/mL penicillin, 100 μg/mL streptomycin sulfate, 50 μM β-mercaptoethanol, and 10 mM non-essential amino acids (referred to as murine T-cell culture medium). For T-cell activation and expansion studies, culture medium was further supplemented with recombinant murine cytokines (all from Novoprotein) as specified in individual experiments: (1) 10 ng/mL mIL-2 alone throughout culture; (2) 10 ng/mL mIL-2 combined with 10 ng/mL mIL-7 from the initiation of culture; (3) 10 ng/mL mIL-2 combined with 10 ng/mL mIL-15 from the initiation of culture; or (4) 10 ng/mL mIL-2 for the first 48 hours, followed by replacement with 10 ng/mL mIL-7 combined with 10 ng/mL mIL-15 for the remainder of the culture period.

### Plasmid construction

All CAR constructs were generated using the pMSCV (murine stem cell virus) retroviral vector backbone (Addgene, #24828). The second-generation CAR architecture consisted of tumor antigen-specific scFv fused to the mouse CD28 hinge and transmembrane domain, followed by the intracellular signaling domains of CD28 and CD3ζ. Three distinct scFvs targeting different tumor antigens were employed: G8.8 (anti-mouse EpCAM), 9F2 (anti-chimeric GPC3), and BM2 (anti-mouse CLDN18.2). Complete sequences of all scFv domains are provided in Supplemental Table S1, http://links.lww.com/HC9/C371. Selected CAR constructs were tagged with mCherry fluorescent protein to enable tracking of transduced cells. For T-cell EpCAM overexpression, the mouse EpCAM overexpression plasmid was constructed using the same pMSCV retroviral vector backbone containing the full-length mouse EpCAM coding sequence.

### Retrovirus production

High-titer, replication-defective retrovirus was produced using Phoenix-Eco or PlatE-Eco packaging cell lines. Cell passage numbers were maintained below 8 generations to ensure optimal packaging efficiency. On day 1, packaging cells were seeded at a density of 1×10^6^ cells per cm^2^ in tissue culture flasks with complete DMEM medium supplemented with 10% FBS and 1% penicillin–streptomycin. On day 0, cell confluence was confirmed to be 70%–85% before proceeding with transfection. The transfection mixture was prepared by combining serum-free DMEM, retroviral transfer plasmid, and pCL-Eco packaging vector at a 3:2 mass ratio (total 40 μg DNA per T175 flask; 24 μg transfer plasmid and 16 μg pCL-Eco), and Mirus TransIT-LT1 transfection reagent (Mirus Bio) or polyethylenimine (PEI) at a 3:1 reagent-to-DNA ratio. The mixture was vortexed thoroughly and incubated for 30 minutes at room temperature. Culture medium was then aspirated from the packaging cells, and the transfection mixture was added and incubated for 1 minute, followed by the addition of fresh DMEM complete medium (25 mL for T175 flasks). On day 1, the viral supernatant was discarded 16–20 hours post-transfection and replaced with 20 mL fresh complete medium. Viral supernatants were harvested at several time points, clarified by centrifugation at 2500 rpm for 10 minutes, and filtered through 0.45 μm filters into 50 mL conical tubes. Fresh medium was added back to the packaging cells after each harvest. Viral supernatants were either used immediately for transduction or aliquoted and stored at −80 °C for future applications.

### Murine CAR-T cell construction

Murine T cells were isolated from single-cell suspensions of splenocytes using the Mouse Pan T Cell Isolation Kit II (Miltenyi Biotec) for positive selection or the MojoSort Mouse CD3 T Cell Isolation Kit (BioLegend) for negative selection (by default). Isolated T cells were seeded at 1×10^6^ cells/mL in murine T cell culture medium and activated with Dynabeads Mouse T-Activator CD3/CD28 beads (Invitrogen) at a 2:1 bead-to-cell ratio or with plate-bound antibodies. For antibody-mediated activation, tissue culture plates were pre-coated with Ultra-LEAF Purified anti-mouse CD3ε (clone 17A2) and anti-mouse CD28 (clone E18) antibodies (BioLegend), each at 5 μg/mL in PBS (400 μL per well for 24-well plates or 2 mL per well for 6-well plates), and incubated overnight at 4 °C. On day 2 post-activation, non-treated 24-well tissue culture plates were coated with recombinant fibronectin fragment RetroNectin (Takara Bio) at 3 μg/cm^2^ in PBS and incubated overnight at 4 °C. On day 3, for bead-mediated activation, cells were harvested and transferred to 1.5 mL microcentrifuge tubes for magnetic bead depletion using the MACSiMAG Separator (Miltenyi Biotec). Tubes were placed in the magnetic field for 2 minutes to allow MACSiBead Particles to adhere to the tube wall. The supernatant containing bead-depleted T cells was carefully transferred to new culture vessels, and the culture volume was doubled with fresh medium. RetroNectin-coated plates were washed twice with PBS, and retroviral supernatants were added (1.5 mL per well for 24-well plates). Plates were centrifuged at 2000*g* for 2 hours at 32 °C to facilitate viral particle loading onto the coated surface. After removing supernatant, activated T cells (5×10^5^ cells in 0.5–1.0 mL per well) were added directly to the virus-loaded wells and allowed to settle for 30 minutes at 37 °C. In selected experiments, polybrene (hexadimethrine bromide; Sigma-Aldrich) was added at a final concentration of 8 μg/mL as a testing reagent to enhance transduction efficiency. Cells were then centrifuged at 1000*g* for 10 minutes at 30 °C and returned to the incubator for overnight culture. Cultures were subsequently maintained at ~1×10^6^ cells/mL with medium replenishment every day. CAR-transduced T cells were cultivated for 7–10 days post-activation before use in functional assays, and CAR expression levels were normalized across experimental groups when necessary. Antibodies used in function assays are showed in supplemental Table S2, http://links.lww.com/HC9/C420.

### In vitro cytotoxicity and cytokine secretion assays

CAR-T cell cytotoxicity was evaluated by detecting live cells defined as Annexin V-negative cells using fluorescence-activated cell sorting (FACS). The cytotoxicity rate for the experimental group was calculated using the formula:


$$\begin{array}{l} \mathrm{Cytotoxicity}\;\mathrm{Rate}=\;(\mathrm{Average}\;\mathrm{percentage}\;\mathrm{of}\;\mathrm{live}\;\mathrm{target}\;\mathrm{cells}\;\mathrm{in}\;\mathrm{Control}\;\mathrm{group}\\ \;\;\;\;\;\;\;\;-\mathrm{Percentage}\;\mathrm{of}\;\mathrm{live}\;\mathrm{cells}\;\mathrm{in}\;\mathrm{experimental}\;\mathrm{group})/\\ \;\;\;\;\;\;\;\;\mathrm{Average}\;\mathrm{percentage}\;\mathrm{of}\;\mathrm{live}\;\mathrm{target}\;\mathrm{cells}\;\mathrm{in}\;\mathrm{Control}\;\mathrm{group}. \end{array}$$

Tumor cell lines or CAR-T cells were stained with carboxyfluorescein succinimidyl ester (CFSE) (Thermo Fisher Scientific) and cultured in murine T-cell culture medium without any added cytokines before co-culture with CAR-T cells. The co-culture duration was determined based on experimental design and conditions. After this period, cells were stained with APC-Annexin V (BioLegend) in combination with either propidium iodide (PI) (BD Biosciences) or 7-AAD (BioLegend) to assess cell death and analyzed using FACS. For the measurement of cytokine secretion, supernatants from the co-culture were tested using mouse IFN-γ ELISA kits (Thermo Fisher Scientific).

For continuous cytotoxicity assay, GPC3-CAR-T cells generated under different cytokine conditions were co-cultured with CellTrace Violet (CTV)-labeled Hepa1-6 cells expressing chGPC3 at a 1:1 effector-to-target ratio. After 4 hours, target cell apoptosis was quantified by 7-AAD and Annexin V staining, with viable tumor cells identified as a 7-AAD^−^Annexin V^−^ double-negative population in the CTV+ gate (Figure 4D). Following the first killing round, CAR-T cells were harvested, counted, re-seeded, and immediately rechallenged with fresh tumor targets to assess sustained cytotoxic function.

### In vivo syngeneic CAR-T cell efficacy assay

This study utilized C57BL/6 mice to assess the efficacy of CAR-T cell therapies against tumor models. For the MC38-EpCAM tumor model, 1 × 10^6^ MC38-EpCAM cells were subcutaneously inoculated into the flanks of the mice on day 12. On day 1, lymphodepletion was induced via intraperitoneal (i.p.) administration of 100 mg/kg cyclophosphamide (CTX). Following lymphodepletion, CAR-T cells specific to EpCAM were infused intravenously (i.v.) on day 0. Throughout the experiment, tumor volume and body weight were recorded daily. For the second model utilizing hepa1-6 chGPC3 cells, 3 × 10^6^ hepa1-6 chGPC3 cells were loaded subcutaneously into C57BL/6 mice on day 7. Lymphodepletion was similarly performed with CTX on day 1. The infusion of chGPC3 CAR-T cells occurred on day 0. Tumor volume and body weight were monitored daily from day 1 to day 21. On day 22, the mice were sacrificed for further analysis of CAR-T cells through flow cytometry (FACS). Throughout the study, tumor volumes were calculated using the formula: V=L×W×W/2 , where *L* is the longest dimension, and *W* is the widest dimension of the tumor. Mice were euthanized either when tumors reached a volume of 2000 mm^3^ or earlier if they exhibited signs of distress or morbidity, in accordance with ethical guidelines.

### ScFv structure and surface electrostatic property prediction

The structure of the single-chain variable fragment (scFv) was predicted using AlphaFold3, and the resulting model was exported as a .pdb file via PyMOL. To analyze the electrostatic properties, the electrostatic potential fields of the charged CAR scFv were visualized using the Swiss-PDBViewer software. The analysis identified the 3 largest surface patches comprised of contiguous positively charged residues, which were assessed using the BindUP web server. In addition, the PCP score, representing the cumulative charge of residues within these prominent patches, was calculated to provide insights into the electrostatic characteristics of the scFv.

### Statistical analysis

Data are presented as means ± standard error of the mean (SEM). Statistical evaluations were conducted using Microsoft Excel and GraphPad Prism version 10.6.0. For multiple comparisons, one-way ANOVA followed by the Dunnett post-test was applied. In vitro experiments were performed in triplicate, with analyses executed using ANOVA or *t* tests as appropriate. A *p*-value of <0.05 was considered statistically significant.

## RESULTS

### Immobilized antibody activation outperforms bead-based stimulation in murine T-cell expansion and homogeneity

Both expansion capacity and retroviral transduction efficiency depend on the T-cell activation state.^12^^,^^25^ We first optimized murine T-cell isolation methods to minimize pre-activation. We compared CD3-positive selection using anti-CD3 magnetic beads versus CD3-negative selection (depleting non-T cells) from lysed splenocytes (Supplemental Figure S1A, http://links.lww.com/HC9/C372). Both methods achieved high T-cell purity (>90% CD3^+^, Supplemental Figures S1B, D, http://links.lww.com/HC9/C372), while negative selection provided a superior yield (Supplemental Figure S1E, http://links.lww.com/HC9/C372). Positive selection with anti-CD3 beads induced high-level premature T-cell activation (Supplemental Figures S1C, F, http://links.lww.com/HC9/C372, *p*<0.01). To avoid confounding pre-activation artifacts, we employed negative selection for all subsequent experiments to ensure a naïve baseline T-cell state.

We next systematically compared immobilized anti-CD3/CD28 antibodies versus CD3/CD28 magnetic beads using saturating concentration with 48-hour activation (Figure 1A). PD-1 expression patterns differed markedly between platforms (Figures 1B, C). Antibody-activated cells showed significantly higher early PD-1 expression, which progressively declined by day 10 to baseline levels. This transient upregulation reflected acute activation signaling rather than terminal exhaustion.^26^ Consistent with this, the robust expansion of antibody-activated cells confirmed that early PD-1 expression did not compromise proliferative capacity.

**FIGURE 1:**
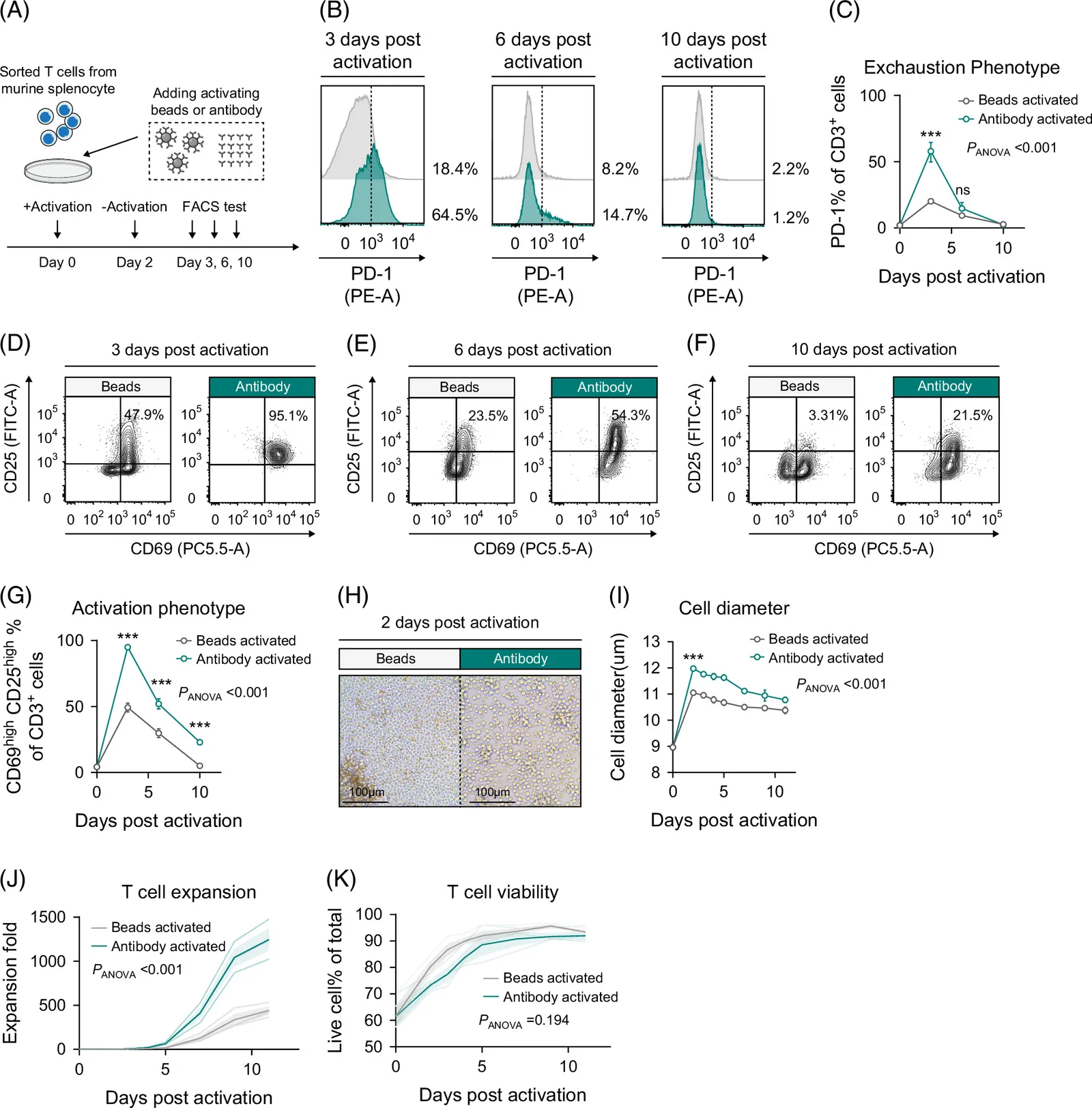
Comparison of antibody-mediated and bead-based T-cell activation platforms. (A) Experimental design schematic. (B) Representative flow cytometry plots of PD-1 expression at the indicated time points. (C) Quantification of PD-1^+^ cells over time. (D–F) Representative flow cytometry plots of CD69 and CD25 expression at the indicated time points. (G) CD69^+^CD25^+^ double-positive cells over time. (H) Representative microscopy images of T cells at day 2 post-activation. (I) Quantification of cell diameter over time. (J) T-cell expansion fold changes over time. (K) Cell viability over time. Data are presented as mean ± SEM (n=3–5 independent experiments). Statistical comparisons between 2 groups at each time point were performed using an unpaired 2-tailed Student *t* test. A 2-way ANOVA was used to assess overall differences across time points and treatment groups, with *p*-values indicated in the text. *p<0.05; **p<0.01; ***p<0.001; ****p<0.0001; ns, not significant. Abbreviations: ANOVA, analysis of variance; FACS, fluorescence-activated cell sorting; PD-1, programmed cell death protein 1; SEM, standard error of the mean.

Activation phenotypes revealed striking differences (Figures 1D–G). The proportion of CD69^high^CD25^high^ double-positive cells peaked at day 3, then progressively declined. At day 3, antibody-activated cells achieved 95.1% CD69^high^CD25^high^ double-positive cells versus only 47.9% for bead-activated cells (*p*<0.001, Figure 1D). This difference remained evident even on day 10 (Figures 1E–G). Antibody-activated cells achieved robust ~1000-fold expansion by day 10, whereas bead-activated cells reached only ~100-fold expansion (Figure 1J). Cell size measurements provided additional evidence of activation status.^27^ Antibody-activated cells were significantly larger throughout the culture period (Figures 1H, I). Both methods maintained comparable high viability throughout the 10-day culture period (Figure 1K), indicating that the poor expansion observed in bead-activated cultures reflected insufficient initial activation rather than increased cell death.

### Optimization of retroviral production and transduction protocols for murine CAR-T manufacturing

Retroviral vector (RVV) production efficiency and transduction optimization are critical bottlenecks in murine CAR-T manufacturing. We systematically optimized the complete workflow from virus packaging to T-cell transduction using a GPC3-targeting CAR construct as a model system. Glypican-3 (GPC3) is a heparan sulfate proteoglycan highly expressed on hepatocellular carcinoma (HCC) but absent from normal adult tissues.^28^ The CAR design incorporated GPC3-specific scFv, CD28 costimulatory domain, and CD3ζ signaling domain (Figures 2A, B).

**FIGURE 2:**
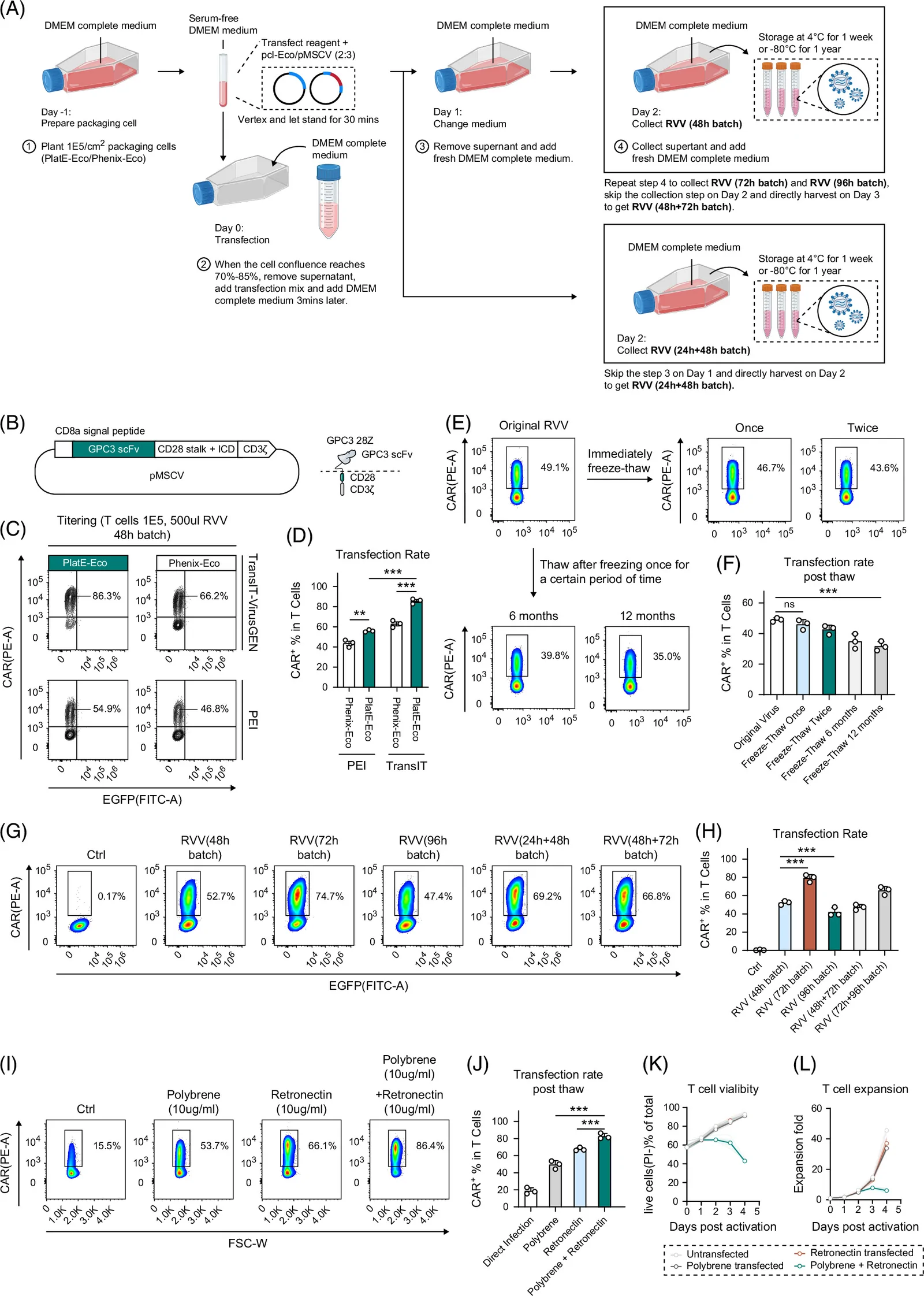
Systematic optimization of retroviral vector production, storage stability, and transduction efficiency for GPC3-CAR-T cell generation. (A) Schematic workflow of retroviral vector packaging and murine T-cell transduction. (B) Plasmid map and structural schematic of chGPC3-targeting CAR construct. (C, D) Comparison of transduction efficiency under different packaging conditions. (E, F) Evaluation of RVV stability under different storage conditions. (G, H) Representative flow cytometry plots and quantification of transduction efficiency of RVV collected at different time points. (I, J) Representative flow cytometry plots and quantification of CAR expression with different transduction enhancement conditions. (K) Changes in cell viability under different transduction conditions. (L) Changes in T-cell expansion under different transduction enhancement conditions. Data are presented as mean ± SEM (n=3 independent experiments). Statistical comparisons between groups were performed using an unpaired 2-tailed Student *t* test. *p<0.05; **p<0.01; ***p<0.001; ****p<0.0001; ns, not significant. Abbreviations: CAR, chimeric antigen receptor; CAR-T, chimeric antigen receptor T-cell; DMEM, Dulbecco’s Modified Eagle Medium; GPC3, glypican-3; RVV, retroviral vector.

Packaging platform selection fundamentally determines viral titer and manufacturing efficiency. We compared packaging reagents and cell lines for RVV production using standardized 48-hour batch collection (Figure 2C). PlatE-Eco cells with TransIT-VirusGEN reagent transfection achieved superior transduction efficiency (Figure 2D).

Viral stability during storage directly impacts manufacturing scalability and workflow flexibility. We assessed RVV transduction capacity following freeze–thaw cycles and prolonged storage. RVV maintained stable transduction efficiency after single or double freeze–thaw cycles (Figures 2E, F). Extended storage at −80 °C preserved viral activity for at least 6 months without significant loss, with modest decline only after 12 months (Figures 2E, F).

Harvest timing is crucial for optimizing viral yield and minimizing losses. We assessed various collection time points and found that a 72-hour batch collection resulted in peak transduction efficiency, significantly higher than the 48-hour or 96-hour batches (Figures 2G, H). However, harvests with double accumulation time (24 h+48 h or 48 h+72 h) did not improve efficiency compared with the single 72-hour collection (Figures 2G, H).

We systematically evaluated polybrene and retronectin as transduction enhancers (Figures 2I–L). While polybrene alone moderately increased transduction, retronectin achieved a higher rate. The combination of polybrene and retronectin resulted in the highest transduction rate (Figures 2I, J), but significantly compromised T-cell quality (Figures 2K, L). This decline in T-cell fitness may be attributed to the membrane toxicity associated with polybrene and the cellular stress arising from an excessive transduction burden.

### Cytokine-mediated modulation of T-cell phenotype and differentiation trajectory

Cytokine selection profoundly influences T-cell differentiation, expansion capacity, and exhaustion status during ex vivo culture.^29^^,^^30^ Following optimization of activation and transduction protocols, we systematically compared 4 clinically relevant cytokine regimens to identify conditions that balance T-cell expansion with fitness preservation: IL-2 alone, IL-2/IL-7, IL-2/IL-15, and IL-7/IL-15 combinations (Figure 3A).

**FIGURE 3:**
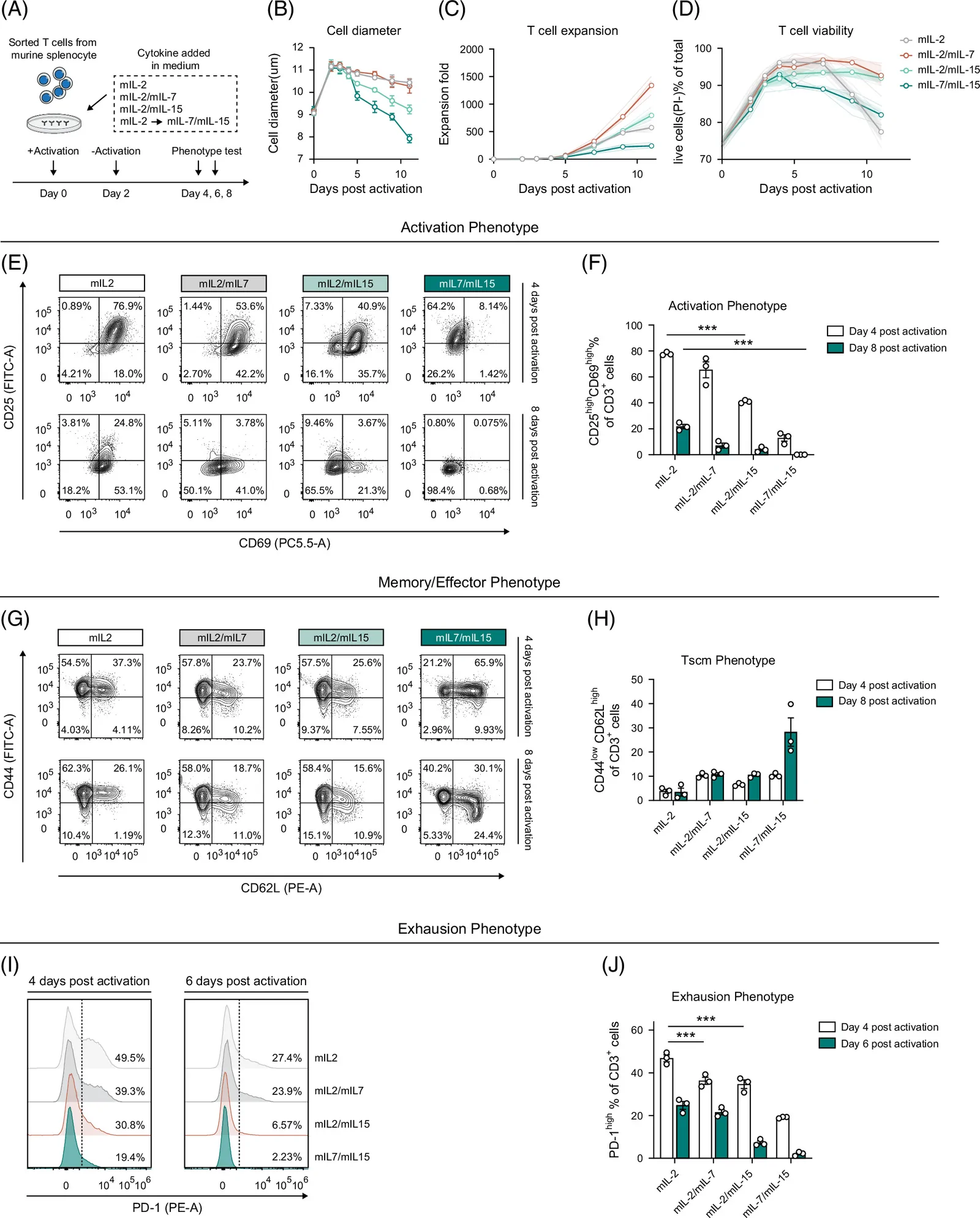
Differential effects of cytokine combinations on T-cell expansion dynamics, phenotypic differentiation, and exhaustion status. (A) Experimental design schematic. (B) Quantification of T-cell diameter over time. (C) T-cell expansion fold changes over time. (D) Cell viability changes over time. (E, F) Representative flow cytometry plots and quantification of CD69 and CD25 expression at days 4 and 8 post-activation. (G, H) Representative flow cytometry plots and quantification of CD44 and CD62L expression at days 4 and 8 post-activation. (I, J) Representative flow cytometry plots and quantification of PD-1 expression at days 4 and 6 post-activation under different cytokine conditions. Data are presented as mean ± SEM (n=3 independent experiments). Statistical comparisons between groups were performed using an unpaired 2-tailed Student *t* test. ***p<0.001; ns, not significant. Abbreviation: PD-1, programmed cell death protein 1.

Cytokine composition significantly influenced T-cell dynamics. IL-2 alone induced rapid cell enlargement, reaching a peak diameter of ~11 μm, while the IL-7/IL-15 combination maintained smaller cell sizes, suggesting preservation of less differentiated states with lower metabolic demand (Figure 3B). The IL-2/IL-7 and IL-2/IL-15 combinations exhibited intermediate cell sizes (Figure 3B). Expansion kinetics revealed that IL-2 and its combinations achieved the highest expansion rates, while the IL-7/IL-15 combination showed reduced expansion (*p*<0.01 vs. IL-2) (Figure 3C). All conditions maintained high viability (>75%) throughout the culture (Figure 3D).

While most conditions exhibited high frequencies of CD25^high^CD69^high^ activated T cells, the IL-7/IL-15 combination showed a decline in activation compared with the other groups (Figures 3E, F). In contrast, IL-2 alone maintained the highest activation signatures, while the IL-2/IL-7 and IL-2/IL-15 combinations showed intermediate activation levels (Figures 3E, F).

Memory phenotype analysis revealed distinct differentiation trajectories under different cytokine conditions (Figures 3G, H). IL-2 alone promoted progressive differentiation toward effector memory T cells (T_EM_, CD62L^−^CD44^+^), with declining stem cell memory T cell (T_SCM_, CD62L^+^CD44^−/ˡᵒ^) populations by day 8. In contrast, IL-7/IL-15 combination preserved central memory and T_SCM_ phenotypes, showing minimal T_EM_ differentiation.

Analysis of PD-1 marker expression revealed distinct phenotypic differences. IL-2 alone induced progressive upregulation of PD-1, with a substantial proportion of PD-1^high^ cells observed by day 4 and sustained levels through day 6 (Figures 3I, J). In contrast, the IL-7/IL-15 combination significantly suppressed PD-1 expression, maintaining a low frequency of PD-1^high^ cells by day 6. The IL-2/IL-7 and IL-2/IL-15 combinations displayed intermediate profiles, with IL-2/IL-15 showing significantly lower PD-1 expression compared with IL-2/IL-7.

### IL-2/IL-7 combination enhances CAR-T cytotoxic capacity and in vivo anti-tumor efficacy

We next evaluated how these distinct phenotypic profiles translate into functional anti-tumor activity. To enable precise tracking and quantification of CAR-T cells in vitro and in vivo, we engineered a bicistronic construct encoding GPC3-specific CAR linked via P2A self-cleaving peptide to mCherry fluorescent reporter (Figures 4A, B).

**FIGURE 4:**
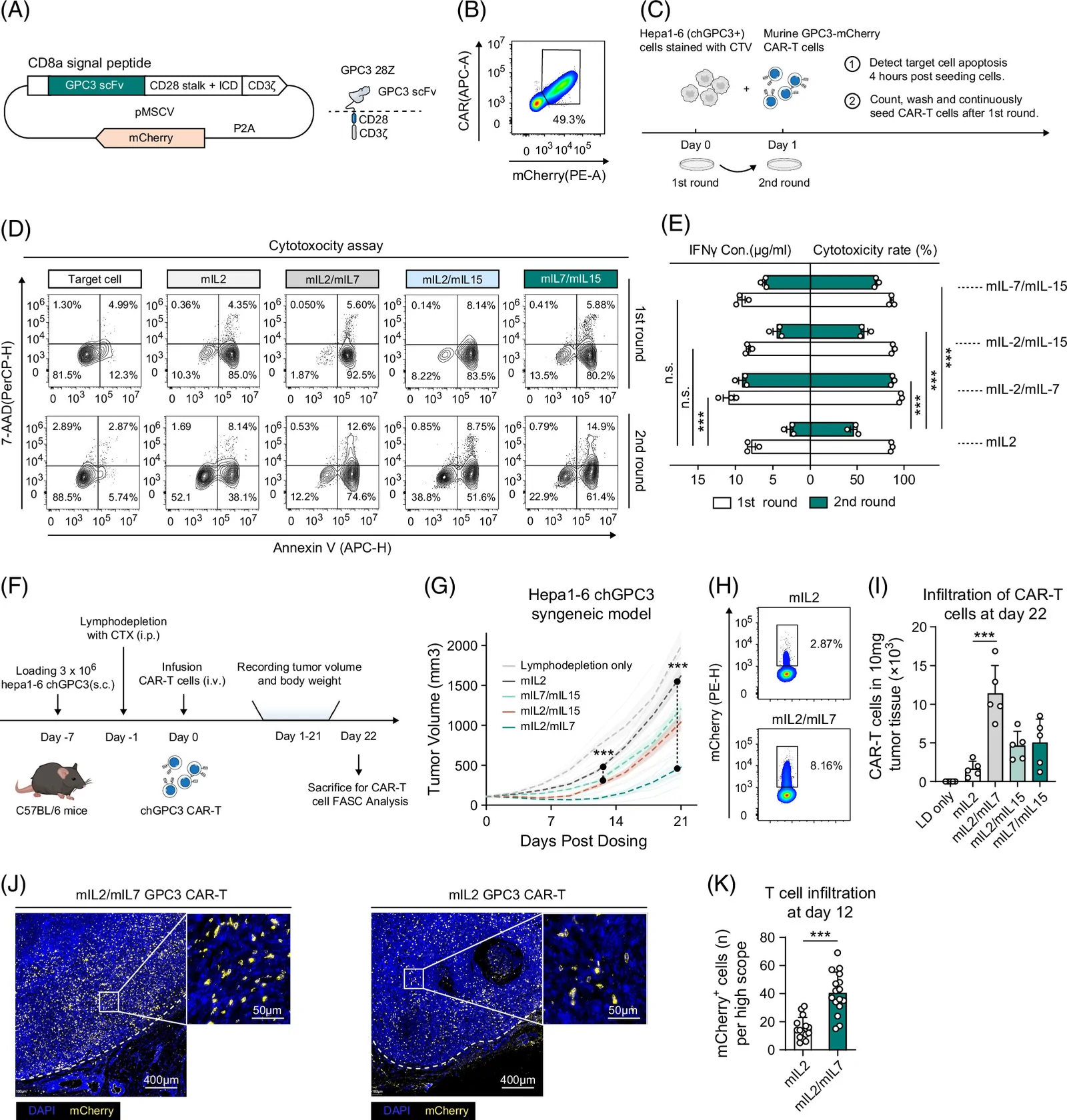
IL-2/IL-7 cytokine combination enhances CAR-T cytotoxic function, tumor infiltration, and therapeutic efficacy in a syngeneic hepatocellular carcinoma model. (A) Plasmid map and structure schematic of bicistronic retroviral vector encoding GPC3-specific CAR linked to mCherry fluorescent reporter via P2A self-cleaving peptide. (B) Representative flow cytometry plots of CAR and mCherry expression. (C) Experimental design for the sequential killing assay. (D) Representative flow cytometry plots showing target cell viability. (E) Quantification of viable tumor cell percentages (left) and IFN-γ secretion levels (right) in culture supernatants. (F) Schematic of the syngeneic subcutaneous tumor model. (G) Tumor growth curves following CAR-T cell therapy. (H) Representative flow cytometry plots showing mCherry^+^ cells among CD3^+^ tumor-infiltrating lymphocytes at day 22 endpoint. (I) Quantification of absolute CAR-T cell counts per 10 mg tumor tissue at experimental endpoint. (J) Representative immunofluorescence microscopy images of tumor sections at day 10 post-CAR-T infusion showing mCherry^+^ CAR-T cell infiltration. (K) Quantification of mCherry^+^ CAR-T cells per high-power field (HPF) in tumor sections at day 10. Data are presented as mean ± SEM (n=5 mice per group for in vivo experiments; n=3 independent experiments for in vitro killing assays). Statistical comparisons between groups were performed using an unpaired 2-tailed Student *t* test for paired comparisons or one-way ANOVA with the Tukey post hoc test for multiple comparisons. *p<0.05; **p<0.01; ***p<0.001; ns, not significant. Abbreviations: CAR, chimeric antigen receptor; CAR-T, chimeric antigen receptor T-cell; CTV, CellTrace Violet; CTX, cyclophosphamide; GPC3, glypican-3; IFN-γ, interferon-gamma; IL, interleukin.

We first assessed cytotoxic capacity using a sequential killing assay that challenges CAR-T cells with multiple rounds of target cell encounter, mimicking the repetitive antigen stimulation encountered in solid tumors (Figure 4C). In the first round of tumor cell targeting, CAR-T cells cultured with IL-2/IL-7 demonstrated a significant reduction in viable tumor cells, outperforming all other conditions, including IL-2 alone, IL-2/IL-15, and IL-7/IL-15 (Figures 4D, E). Remarkably, in the second round of sequential killing, IL-2/IL-7 CAR-T cells retained robust cytotoxicity, while the other conditions exhibited a notable decline in their ability to eliminate tumor cells. Consistent with enhanced cytotoxic function, IL-2/IL-7 CAR-T cells secreted significantly higher levels of IFN-γ in both the first and second rounds compared with other conditions, reflecting preserved effector function and activation capability (Figure 4E).

To evaluate therapeutic efficacy in immunocompetent settings, we established a syngeneic Hepa1-6 chGPC3 subcutaneous tumor model in C57BL/6 mice (Figure 4F). IL-2/IL-7 CAR-T cells induced profound tumor regression, significantly superior to IL-2 alone (*p*<0.001, Figure 4G). We assessed CAR-T cell persistence and tumor infiltration at the experimental endpoint. Flow cytometric analysis of tumor-infiltrating lymphocytes revealed that IL-2/IL-7 conditioning led to CAR-T cells with superior in vivo persistence (Figures 4H, I). To capture the earlier dynamics of CAR-T cell tumor infiltration, we conducted an independent experiment with tissue harvesting at day 10 post-infusion (Figure 4J). Immunofluorescence microscopy of tumor sections confirmed a dramatically enhanced infiltration of mCherry^+^ CAR-T cells in the IL-2/IL-7 condition (Figures 4J, K).

Collectively, these data demonstrate that IL-2/IL-7 cytokine combination generates CAR-T cells with superior cytotoxic capacity, sustained effector function upon serial antigen encounter, enhanced tumor infiltration, prolonged in vivo persistence, and improved therapeutic efficacy.

### Positively charged patches on CAR scFv drive tonic signaling and exhaustion through electrostatic-mediated CAR clustering

While optimizing CAR-T manufacturing protocols, we encountered a persistent challenge with G8.8 scFv-based CAR targeting mouse EpCAM.^31^ Despite successful transduction and CAR expression, G8.8 CAR-T cells exhibited severely impaired expansion and premature exhaustion during ex vivo culture. To investigate this phenomenon, we compared G8.8 EpCAM-targeting CAR with 2 other well-characterized CARs: 9F2 targeting GPC3 and BM2 targeting Claudin18.2, all sharing identical CD28–CD3ζ signaling architecture (Figure 5A).

**FIGURE 5:**
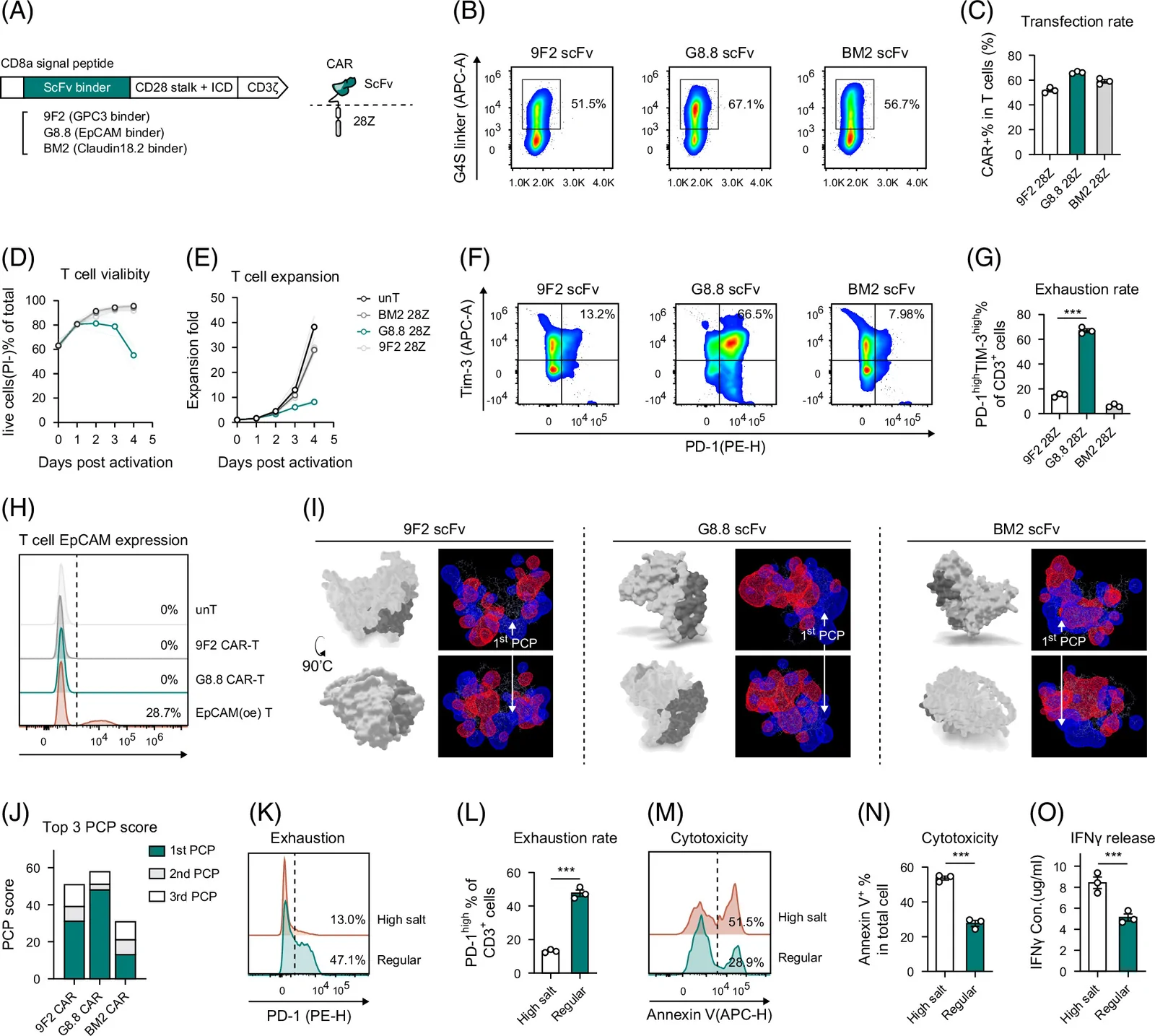
Positively charged patches on G8.8 scFv drive CAR clustering and tonic signaling-induced exhaustion, which can be rescued by high-salt culture conditions. (A) Schematic comparison of 3 CAR constructs. (B) Representative flow cytometry plots showing CAR expression on transduced T cells. (C) Quantification of CAR^+^ cell frequencies post-transduction. (D) T-cell expansion fold changes over time. (E) Cell viability changes over time. (F) Representative flow cytometry plots and quantification of PD-1 and Tim-3 expression at day 5 post-activation. (H) Flow cytometric analysis of EpCAM expression on untransduced T cells (unT), EpCAM-overexpressing T cells, and CAR-T cells. (I) AlphaFold3-predicted structures of 9F2, G8.8, and BM2 scFvs with surface electrostatic potential mapping using the Swiss-PdbViewer software. Blue regions indicate positively charged patches (PCPs), red regions indicate negatively charged areas. Note the prominent large PCP (1st PCP). (J) Quantification of PCP scores for the 3 scFvs. The total PCP score represents the sum of residues within the 3 largest continuous positively charged patches. 1st PCP indicates the number of residues in the largest single positively charged patch. (K) Representative flow cytometry plots showing PD-1 expression on G8.8 CAR-T cells cultured in regular medium versus high-salt medium (supplemented with 50 mM NaCl) at day 5 post-activation. (L) Quantification of PD-1^high^ exhausted T cells under different culture conditions. (M) Representative flow cytometry plots showing target cell apoptosis (7-AAD and Annexin V staining) following 4-hour co-culture with G8.8 CAR-T cells cultured under regular or high-salt conditions. (N) Quantification of Annexin V^−^ live tumor cells. (O) IFN-γ secretion levels in culture supernatants following target cell encounter. Data are presented as mean ± SEM (n=3 independent experiments). Statistical comparisons between groups were performed using an unpaired 2-tailed Student *t* test for paired comparisons or one-way ANOVA with the Tukey post hoc test for multiple comparisons. **p<0.01; ***p<0.001; ns, not significant. Abbreviations: ANOVA, analysis of variance; CAR, chimeric antigen receptor; CAR-T, chimeric antigen receptor T-cell; EpCAM, epithelial cell adhesion molecule; IFN-γ, interferon-gamma; PD-1, programmed cell death protein 1; scFv, single-chain variable fragment.

Initial characterization revealed comparable transduction efficiencies across all three CAR constructs (Figures 5B, C). However, striking differences emerged during ex vivo expansion. While 9F2 and BM2 CAR-T cells achieved robust 30–40-fold expansion by day 4 post-activation, G8.8 CAR-T cells showed severely compromised proliferative capacity with only ~10-fold expansion (Figure 5E). This expansion defect correlated with progressive viability decline, as G8.8 CAR-T cells dropped to ~60% viability by day 5, compared with >85% viability maintained by 9F2 and BM2 CAR-T cells (Figure 5D). Flow cytometric analysis revealed that G8.8 CAR-T cells developed profound exhaustion (Figures 5F, G).

We first hypothesized that EpCAM expression on activated T cells might trigger fratricide and exhaustion. However, flow cytometric analysis excluded this possibility. While EpCAM-overexpressing control T cells showed robust surface expression, neither untransduced T cells nor 9F2, G8.8, or BM2 CAR-T cells exhibited detectable EpCAM expression (Figure 5H).

Recent studies have demonstrated that positively charged patches (PCPs) on CAR scFv surfaces mediate spontaneous CAR clustering through electrostatic interactions, driving antigen-independent tonic signaling and T-cell exhaustion.^13^ To investigate whether differential surface electrostatics could explain G8.8 CAR-T dysfunction, we performed structural modeling using AlphaFold3 and calculated electrostatic potential distributions using the Swiss-PdbViewer software (Figure 5I). G8.8 scFv displayed a prominent positively charged patch (indicated as 1st PCP) substantially larger and more intense than those observed on 9F2 or BM2 scFvs, which showed relatively even charge distribution across their surfaces.

The PCP score represents the sum of residues within the 3 largest positively charged patches on the scFv surface.^13^ All 3 ScFv structures exhibited a moderate total PCP score (Figure 5J). However, we noticed that G8.8’s dominant PCP contained 49 residues, nearly double that of 9F2 (26 residues) and BM2 (22 residues). We propose that this highly concentrated positive charge may create intense local electrostatic fields capable of driving CAR clustering and sustained tonic signaling even when the total charge is not dramatically elevated.

We then tested whether neutralizing electrostatic interactions could rescue G8.8 CAR-T function. We cultured G8.8 CAR-T cells in high-salt medium (standard medium supplemented with 50 mM NaCl) immediately after transduction. Increased ionic strength shields surface charges and disrupts electrostatic-driven protein–protein interactions without affecting antigen–receptor binding affinity. Remarkably, high-salt culture profoundly improved G8.8 CAR-T phenotype and function (Figures 5K–O). By day 5 post-activation, G8.8 CAR-T cells cultured in high-salt conditions exhibited significantly reduced signs of exhaustion (Figures 5K, L). This prevention of exhaustion was accompanied by a notable restoration of functionality. In vitro cytotoxicity assays demonstrated that high-salt cultured G8.8 CAR-T cells achieved a markedly higher rate of target cell apoptosis compared with those cultured in regular medium (Figures 5M, N). Correspondingly, high-salt cultured G8.8 CAR-T cells secreted significantly higher levels of IFN-γ upon encountering target cells (Figure 5O).

We concluded that excessive positive charge concentration on CAR scFv surfaces—specifically, large 1st PCP scores—drives spontaneous CAR clustering, sustained tonic signaling, and premature exhaustion during ex vivo culture.

### Rational engineering of G8.8 scFv electrostatics eliminates tonic signaling and restores CAR-T cell function

We next sought to rationally engineer an improved variant with optimized surface electrostatics while preserving antigen-binding specificity. Detailed sequence annotation of G8.8 scFv revealed that the 1st PCP, comprising 49 positively charged residues, was distributed across both the heavy and light chain framework regions (FRs) and the complementarity-determining regions (CDRs). (Figure 6A, blue-highlighted residues). Based on AlphaFold3 structural predictions and electrostatic potential mapping, we identified several lysine (K) residues within framework regions that were primary contributors to the excessive positive charge concentration. These lysine residues were strategically substituted with glutamine (Q), a polar but uncharged amino acid with similar structural properties, generating the charge-neutralized variant designated Opti-G8.8 (Figure 6B, red-labeled residues). All 6 CDRs remained completely unchanged to preserve EpCAM-binding affinity and specificity.

**FIGURE 6:**
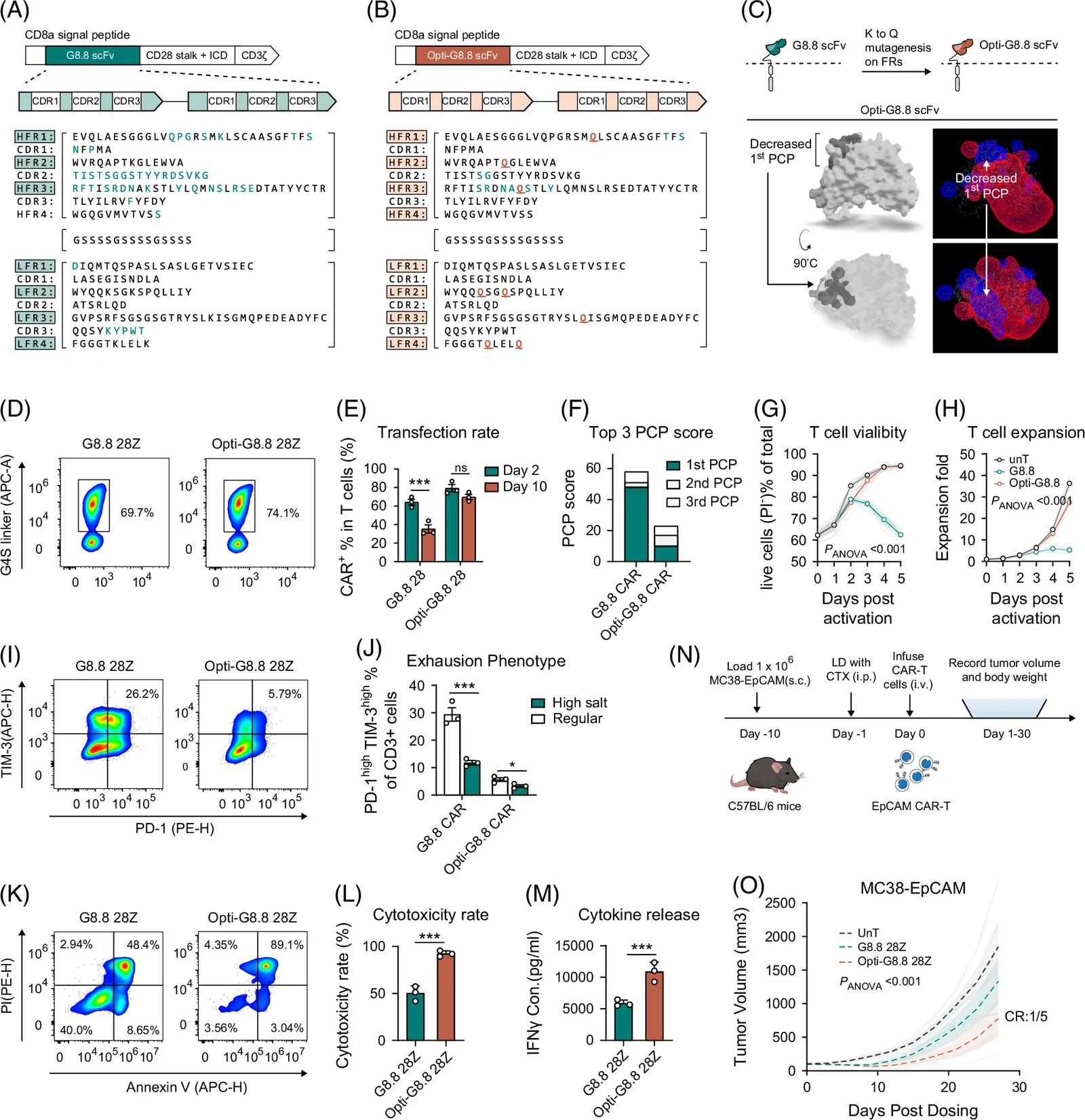
Rational charge optimization of G8.8 scFv eliminates tonic signaling and restores CAR-T cell fitness and therapeutic efficacy. (A) Amino acid sequence of G8.8 scFv showing heavy chain variable region (VH) and light chain variable region (VL) connected by a (G_4_S)_3_ linker. Green-highlighted residues indicate amino acids within the 1st positively charged patch (PCP). CDR regions (CDR1, CDR2, CDR3) and framework regions (FR1–FR4) are annotated. (B) Sequence comparison between G8.8 and Opti-G8.8 scFv. Red-labeled residues (Q) indicate lysine-to-glutamine (K→Q) substitution sites designed to neutralize excessive positive charge. Blue-highlighted residues show the substantially reduced 1st PCP in Opti-G8.8 compared with G8.8. (C) AlphaFold3-predicted 3-dimensional structures of G8.8 (left) and Opti-G8.8 (right) scFvs with surface electrostatic potential mapping. Blue regions represent positively charged patches, red regions indicate negatively charged areas. (D) Representative flow cytometry plots showing CAR expression post-transduction. (E) Quantification of CAR^+^ cell frequencies. (F) Quantitative comparison of PCP scores between G8.8 and Opti-G8.8 scFvs. Total PCP score represents the sum of residues within the 3 largest continuous positively charged patches; 1st PCP indicates the number of residues in the largest single patch. (G) T-cell expansion fold changes over time. (H) Cell viability changes over time. (I, J) Representative flow cytometry plots and quantification of PD-1 and Tim-3 expression on unT, G8.8 CAR-T, and Opti-G8.8 CAR-T cells at day 6 post-activation without antigen stimulation. (K, L) Representative flow cytometry plots and quantification of target cell apoptosis. (M) IFN-γ secretion levels in culture supernatants. (N) Schematic of in vivo therapeutic efficacy study design. (O) Tumor growth curves over 30 days post-CAR-T infusion. Individual tumor volumes are shown as thin lines; mean ± SEM are shown as thick lines with shaded areas (n=5 mice per group). Data in panels E, F, G, H, J, L, and M are presented as mean ± SEM from at least 3 independent experiments. Statistical comparisons were performed using an unpaired 2-tailed Student *t* test for paired comparisons or one-way ANOVA with the Tukey post hoc test for multiple comparisons. Tumor growth curves were analyzed by repeated measures two-way ANOVA. *p<0.05; **p<0.01; ***p<0.001; ns, not significant. Abbreviations: ANOVA, analysis of variance; CAR, chimeric antigen receptor; CAR-T, chimeric antigen receptor T-cell; CDR, complementarity-determining region; IFN-γ, interferon-gamma; PD-1, programmed cell death protein 1; scFv, single-chain variable fragment.

Quantitative PCP scoring confirmed the effectiveness of this optimization strategy (Figures 6C, F). Flow cytometric analysis confirmed that charge optimization did not impair CAR surface expression, with comparable transduction efficiencies and expression levels between G8.8 and Opti-G8.8 constructs (Figures 6D, E). Most importantly, Opti-G8.8 optimization completely rescued the severe expansion defect associated with G8.8 CAR-T cells. Following activation, Opti-G8.8 CAR-T cells achieved ~30-fold expansion by day 5 post-activation, nearly identical to untransduced T cells and superior to G8.8 CAR-T cells (Figure 6H, *p*<0.001). This restoration was accompanied by complete rescue of cell viability (Figure 6G, *p*<0.001). Phenotypic analysis at day 6 post-activation revealed that charge optimization significantly prevented the upregulation of exhaustion markers in the absence of antigen stimulation (Figures 6I, J). This indicates that rational charge optimization effectively eliminates tonic signaling-driven exhaustion without compromising CAR expression. In vitro cytotoxicity assays demonstrated that Opti-G8.8 CAR-T cells achieved a significantly higher level of tumor cell apoptosis compared with G8.8 CAR-T cells (Figures 6K, L). Consistent with this enhanced cytotoxicity, Opti-G8.8 CAR-T cells secreted substantially more IFN-γ compared with their counterparts (Figure 6M).

Furthermore, we evaluated therapeutic efficacy in an immunocompetent syngeneic MC38-EpCAM tumor model. Opti-G8.8 CAR-T therapy induced substantial tumor regression, while G8.8 CAR-T cells only provided a modest delay in tumor growth (Figure 6O). Collectively, these studies illustrate that the rational reduction of surface positive charge density through targeted amino acid substitutions represents a useful engineering strategy to eliminate tonic signaling, restore normal T-cell expansion, prevent exhaustion, and enhance therapeutic efficacy.

## DISCUSSION

Our findings demonstrate that the integration of optimized T-cell activation methods, cytokine supplementation strategies, and structure-based electrostatic surface engineering synergistically enhances CAR-T cell fitness, mitigates exhaustion, and substantially improves therapeutic efficacy in primary hepatocellular carcinoma and colorectal liver metastasis models. These advances address critical barriers that have limited CAR-T translation to solid tumors and provide actionable strategies with direct translational potential.^32^

This study demonstrated that immobilized anti-CD3/CD28 antibody activation outperforms bead-based stimulation in murine CAR-T manufacturing. This difference likely arises from the consistent signal delivery of immobilized antibodies versus the variable contact dynamics in bead-based systems.^33^ The transient early upregulation of PD-1 with antibody activation indicates acute activation signaling rather than exhaustion, supported by sustained long-term expansion and viability.^26^

The cytokine milieu profoundly shaped CAR-T cell phenotype and therapeutic potential. While IL-2 provides the potent proliferative signaling necessary for initial T-cell activation and rapid biomass expansion, its prolonged use can drive terminal differentiation and exhaustion. In contrast, the transition to IL-7 and IL-15 promotes long-term persistence and the preservation of a stem-like phenotype.^17^^,^^34^ Critically, IL-7 co-stimulation reduced exhaustion marker expression even during prolonged ex vivo culture, suggesting active prevention of differentiation toward terminal effector states. These findings support a strategic shift toward homeostatic cytokine-based manufacturing protocols that balance expansion requirements with preservation of stem-like properties essential for in vivo persistence and function.

Our electrostatic optimization strategy addresses a fundamental yet underappreciated liability in CAR design: antigen-independent tonic signaling driven by spontaneous CAR clustering mediated by excessive positive charge patches. The G8.8 scFv exemplifies this problem. Rational charge neutralization completely rescues T-cell expansion, viability, and effector function. The dramatic therapeutic superiority of Opti-G8.8 in the immunocompetent MC38-EpCAM model validates that electrostatic-mediated tonic signaling represents a correctable design flaw rather than an inherent limitation of certain scFvs.^35^ This structure-guided engineering approach offers broad applicability, as computational prediction of PCPs and targeted charge optimization can be applied prospectively to any scFv during CAR development, potentially preventing exhaustion liabilities before clinical translation.

The use of immunocompetent syngeneic mouse models throughout this study provides critical advantages over conventional immunodeficient xenograft systems. Syngeneic models enable assessment of CAR-T cells within an intact immune system, capturing physiologically relevant interactions including cytokine networks, immune cell crosstalk, and tumor microenvironment (TME) remodeling that profoundly influence therapeutic outcomes.^36^

Given the clinical complexity of liver malignancies, achieving superior therapeutic outcomes requires not only potent genetic constructs but also advanced manufacturing and optimization strategies.^37^ Several aspects of our findings warrant further investigation. First, while we demonstrate superior performance of IL-7 supplementation, the optimal cytokine formulation may vary depending on target antigen, tumor type, and signaling domain composition. Second, our electrostatic optimization focused on neutralizing excessive positive charge, but the functional consequences of negative charge patches and optimal overall net charge remain incompletely defined. Comprehensive charge landscape engineering, potentially incorporating machine learning approaches, may enable further refinement.

In conclusion, this study establishes that systematic optimization of CAR-T manufacturing processes and structure-based engineering to eliminate tonic signaling synergistically enhances therapeutic efficacy against hepatic malignancies in immunocompetent models. By addressing T-cell exhaustion through multiple complementary mechanisms—optimized initial activation, memory-preserving cytokine conditions, and elimination of antigen-independent signaling—we demonstrate substantial improvements in CAR-T expansion, persistence, and tumor control.

## Supplementary Material
