# Supplementary material for: Manufacturing and electrostatic surface optimization of CAR-T cells improve therapeutic efficacy in liver malignancies
Source: Hepatol Commun. 2026 Jul 15;10(8):e00982. doi: 10.1097/HC9.0000000000000982 (PMC13671602; doi:10.1097/HC9.0000000000000982)
Supplement: Supplementary file 1 [file hc9-10-e00982-s002.pdf]

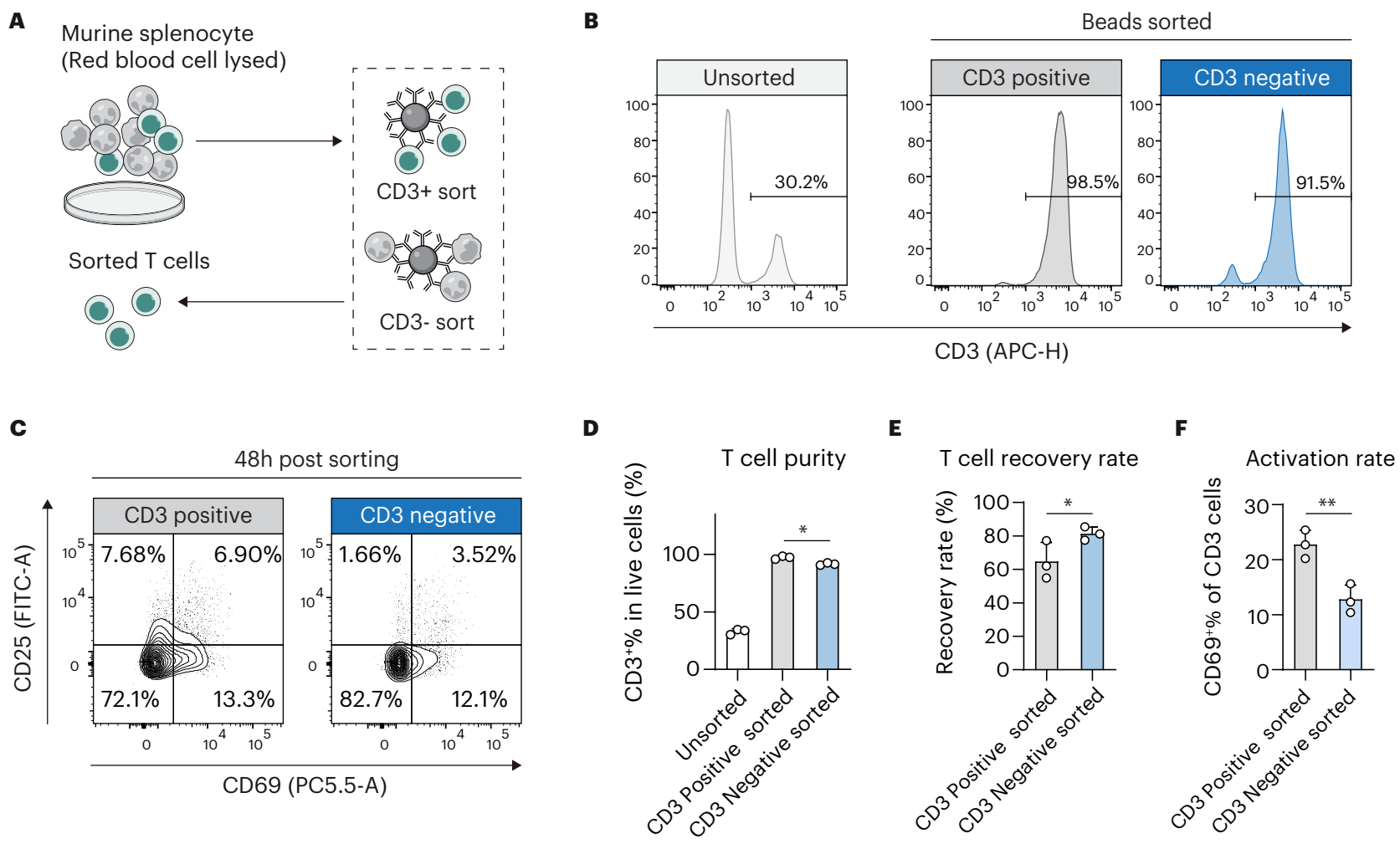

Supplementary Figure 1. Comparison of CD3-negative and CD3-positive selection methods for murine T cell isolation and their impact on T cell activation status. (A) Schematic diagram illustrating. (B) Representative flow cytometry plots showing CD3+ T cell purity. (C) Representative flow cytometry and quantification of activation markers CD69 and CD25 expression 48 hours post-sorting without any deliberate stimulation. (D) Quantification of T cell purity (CD3+ percentage within live cells) for both isolation methods. (E) T cell recovery rate comparison between CD3-positive and CD3-negative selection methods. (F) Quantification of CD69+ activated T cells at 48 hours post-sorting without deliberate stimulation. Data are presented as mean ± SEM from three independent experiments. Statistical comparisons were performed using unpaired two-tailed Student's t-test. *P < 0.05; **P < 0.01.
