# Supplementary material for: Manufacturing and electrostatic surface optimization of CAR-T cells improve therapeutic efficacy in liver malignancies
Source: Hepatol Commun. 2026 Jul 15;10(8):e00982. doi: 10.1097/HC9.0000000000000982 (PMC13671602; doi:10.1097/HC9.0000000000000982)
Supplement: Supplementary file 2 [file hc9-10-e00982-s003.pdf]

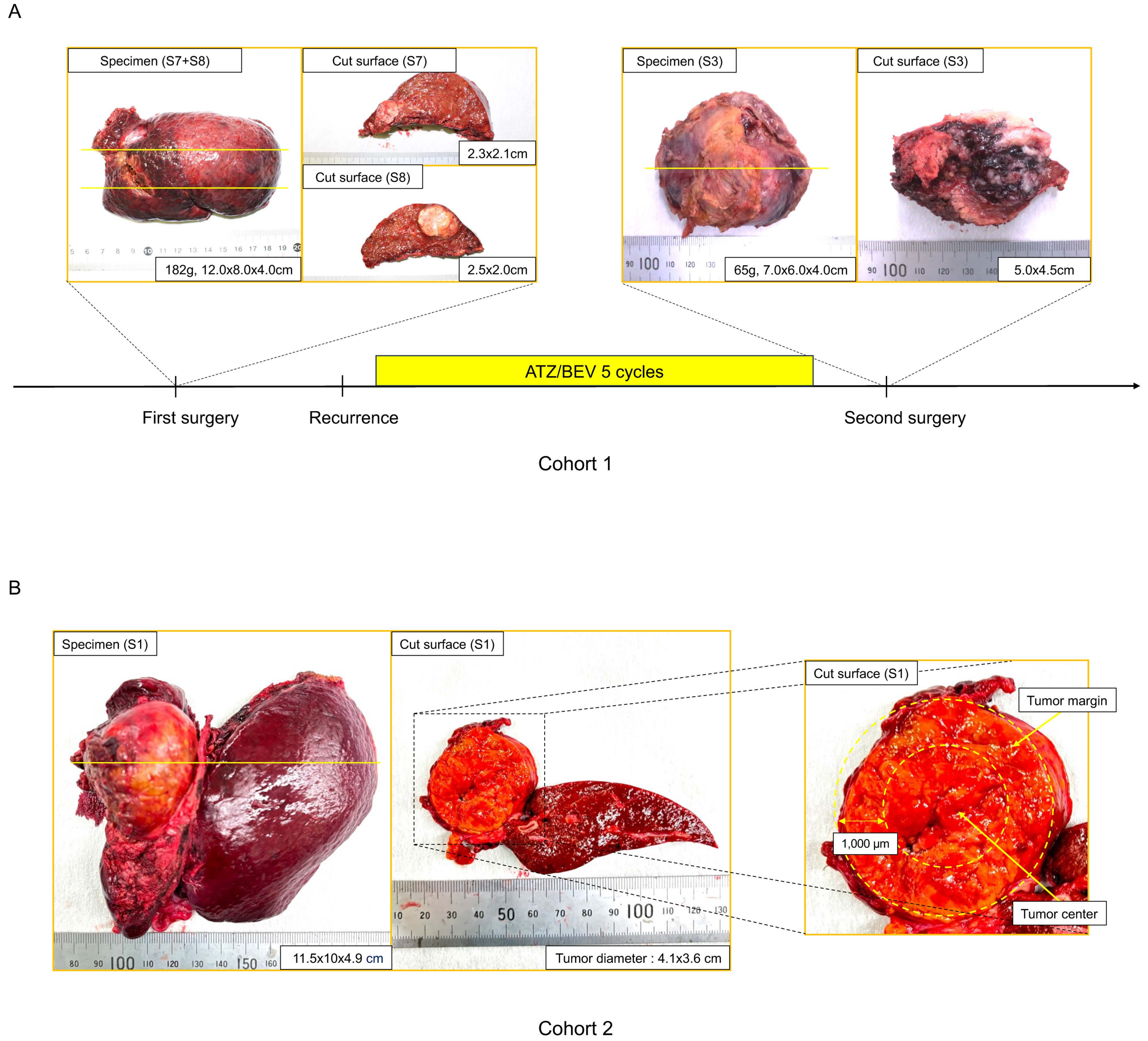

Supplementary Figure. 1 Clinical course, surgical specimens, and sampling strategy of a recurrent hepatocellular carcinoma case.

(A) The diagram illustrates the treatment course of a patient with hepatocellular carcinoma (HCC) who developed intrahepatic recurrence following initial hepatectomy. The primary surgery involved resection of tumors located in hepatic segments 7 and 8. Postoperatively, recurrence in segment 3 was detected, for which five cycles of atezolizumab plus bevacizumab (ATZ/BEV) were administered, resulting in stable disease. A second hepatectomy was subsequently performed to remove the recurrent lesion. Macroscopic views of the resected specimens, including external appearance and cut surfaces with measured tumor sizes, are shown.

(B) Hepatectomy was performed for a HCC located in segment 1 following treatment with ATZ/BEV. For downstream single-cell analysis, the tumor edge was defined as the 1,000 μm region extending inward from the interface between non-tumorous liver parenchyma and tumor tissue, while the area further inside was designated as the tumor center. Representative macroscopic images of the whole specimen and the cut surface, including the annotated tumor edge and tumor center regions, are shown.

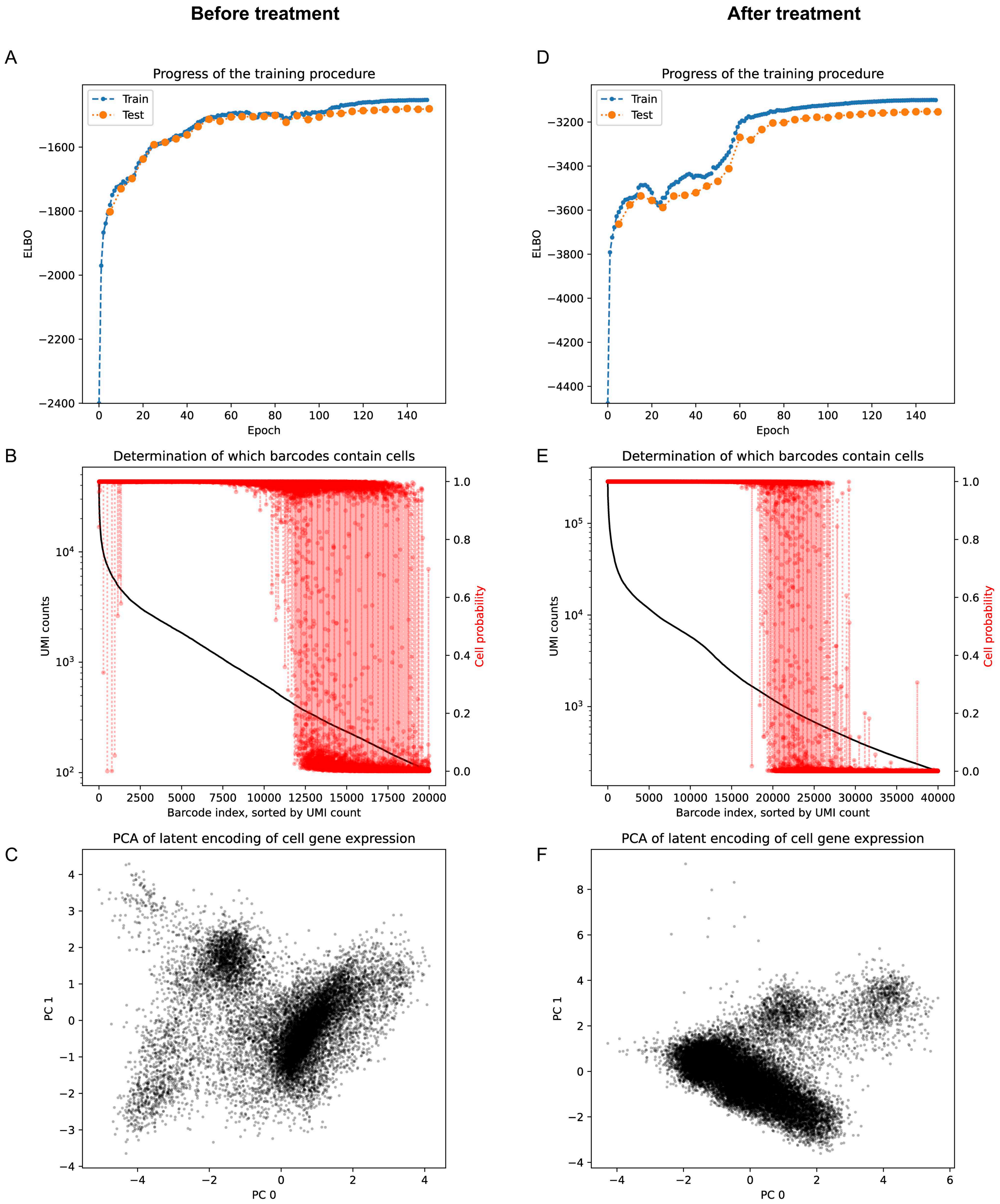

Supplementary Figure. 2 CellBender ambient RNA removal quality control for before and after treatment samples.

CellBender (v0.2.1) diagnostic output for the pre-treatment (before) and post-treatment (after) samples. Each panel set contains three plots. (A, D) Training curve showing the Evidence Lower BOund (ELBO) over 150 epochs for the training (blue) and test (orange) sets; convergence of both curves indicates stable model fitting. (B, E) Barcode rank plot showing UMI counts per barcode (black line, left axis) and CellBender-estimated cell probability (red dots, right axis) as a function of barcode index sorted by decreasing UMI count; barcodes with high cell probability (red, ~1.0) are classified as cell-containing droplets, whereas low-probability barcodes represent empty droplets used to estimate the ambient RNA profile. The before sample contained approximately 12,500 cell barcodes, while the after sample contained approximately 17,500 cell barcodes. (C, F) PCA of the CellBender latent representation of decontaminated cell gene expression, visualizing the transcriptional diversity of recovered cells after ambient RNA correction.

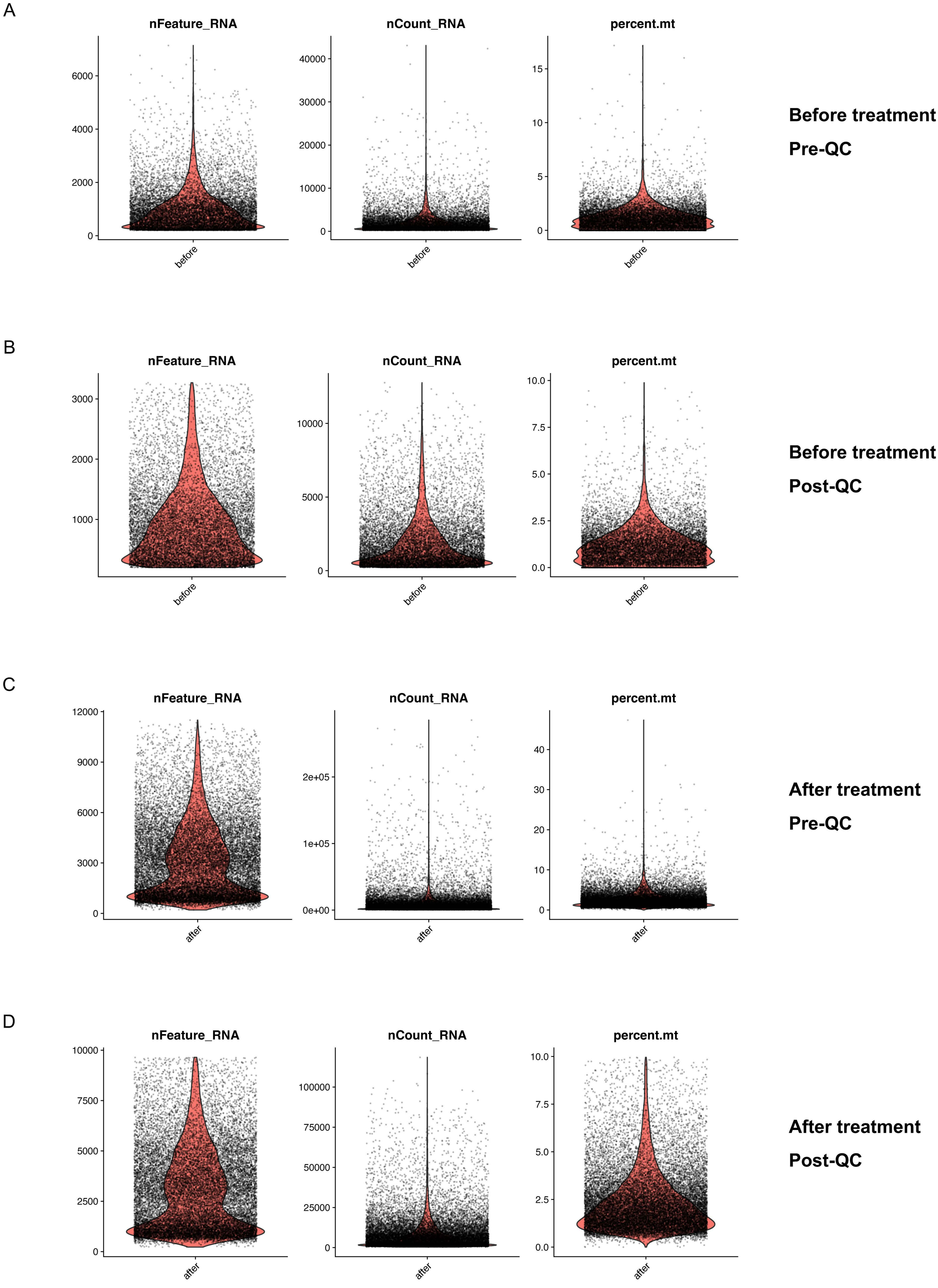

Supplementary Figure. 3 Quality control metrics of single-cell RNA-sequencing samples.
Violin plots show the quality control (QC) results of tumors before and after ATZ/BEV therapy. Samples obtained before treatment are shown before QC (A) and after QC (B), whereas samples obtained after treatment are shown before QC (C) and after QC (D). The quality control metrics include the number of detected genes per cell (nFeature_RNA), total RNA counts per cell (nCount_RNA), and the percentage of mitochondrial transcripts (percent.mt).

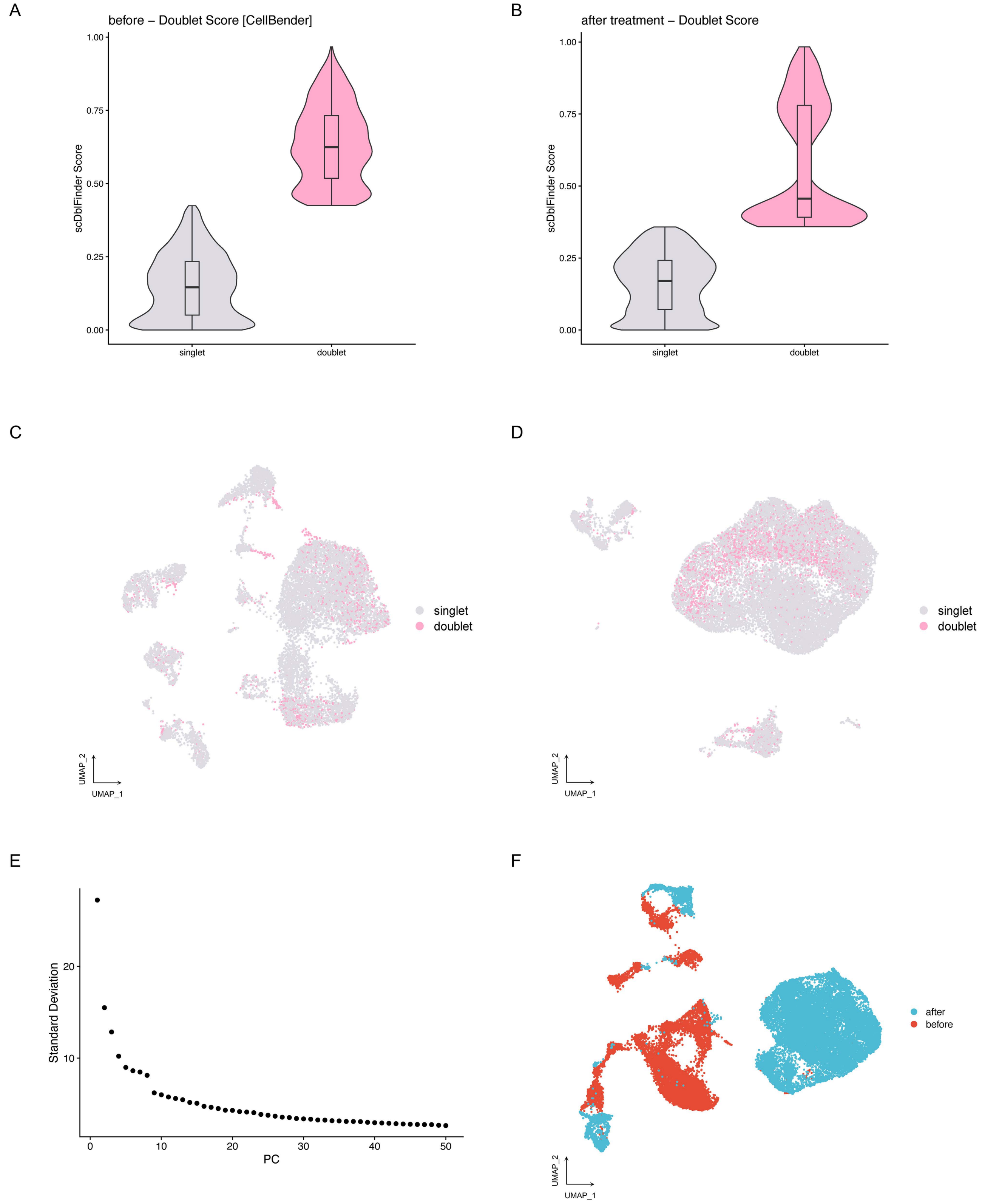

Supplementary Figure. 4 Doublet detection and batch-effect assessment in scRNA-seq data before downstream integration.

(A, B) Violin plots of scDblFinder scores. Doublet probability scores assigned by scDblFinder (v1.22.0) to each barcode of samples before and after treatment. Barcodes classified as singlets (grey) show consistently low scores (median ~0.15), whereas barcodes classified as doublets (pink) show markedly higher scores (median ~0.6 in before; ~0.45 in after), demonstrating clear separation between the two populations. The overlaid box plots indicate the median and interquartile range. Barcodes classified as doublets by scDblFinder were excluded before downstream integration and clustering. Residual mixed-profile clusters identified during subsequent lineage-specific subclustering were evaluated separately and excluded where applicable before downstream analyses. (C, D) UMAP visualization of doublet distribution prior to removal. UMAP embedding computed from the top 30 principal components of normalized gene expression (before Harmony batch correction). Each dot represents a single barcode, colored by scDblFinder classification: singlets (grey) and doublets (pink). (E) The contribution of the top 50 principal components (PCs), calculated from SCTransform-normalized gene expression data of merged cells, is shown. The variance explained declined sharply from PC1 to around PC10 and then decreased more gradually. Based on this distribution, the top 40 PCs were used for downstream analysis. (F) UMAP of the merged cells was computed from the top 40 PCs before batch correction. Cells were clearly separated between the before and after groups, indicating a marked batch effect. Harmony correction was therefore applied before cell type annotation.

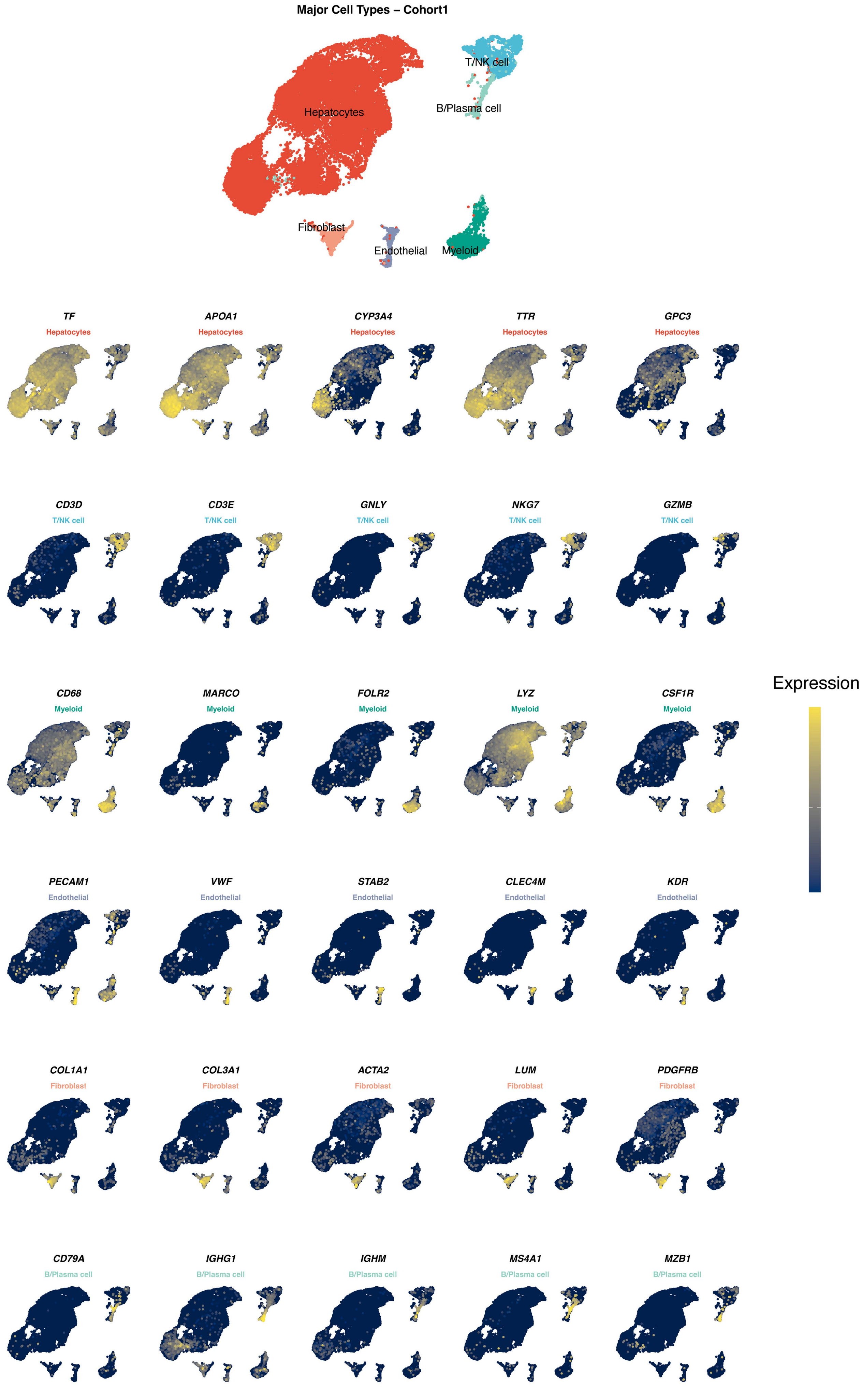

Supplementary Figure. 5 Expression of canonical cell-type marker genes in hepatocellular carcinoma samples before and after treatment.

UMAP visualization of QC-filtered high-quality single cells from before-treatment and after-treatment liver tumor samples (n = 27,850 cells), colored by annotated major cell type. Feature plots show normalized expression of canonical marker genes for each cell population: Hepatocytes (TF, APOA1, CYP3A4, TTR, GPC3); T/NK cells (CD3D, CD3E, GNLY, NKG7, GZMB); Myeloid cells (CD68, MARCO, FOLR2, LYZ, CSF1R); Endothelial cells (PECAM1, VWF, STAB2, CLEC4M, KDR); Fibroblasts (COL1A1, COL3A1, ACTA2, LUM, PDGFRB); B/Plasma cells (CD79A, IGHG1, IGHM, MS4A1, MZB1). Color scale represents normalized expression level (cividis palette; low to high). Color scales are shown individually for each gene and should not be compared directly across markers. Cell type labels in feature plot subtitles are color-coded to match the UMAP.

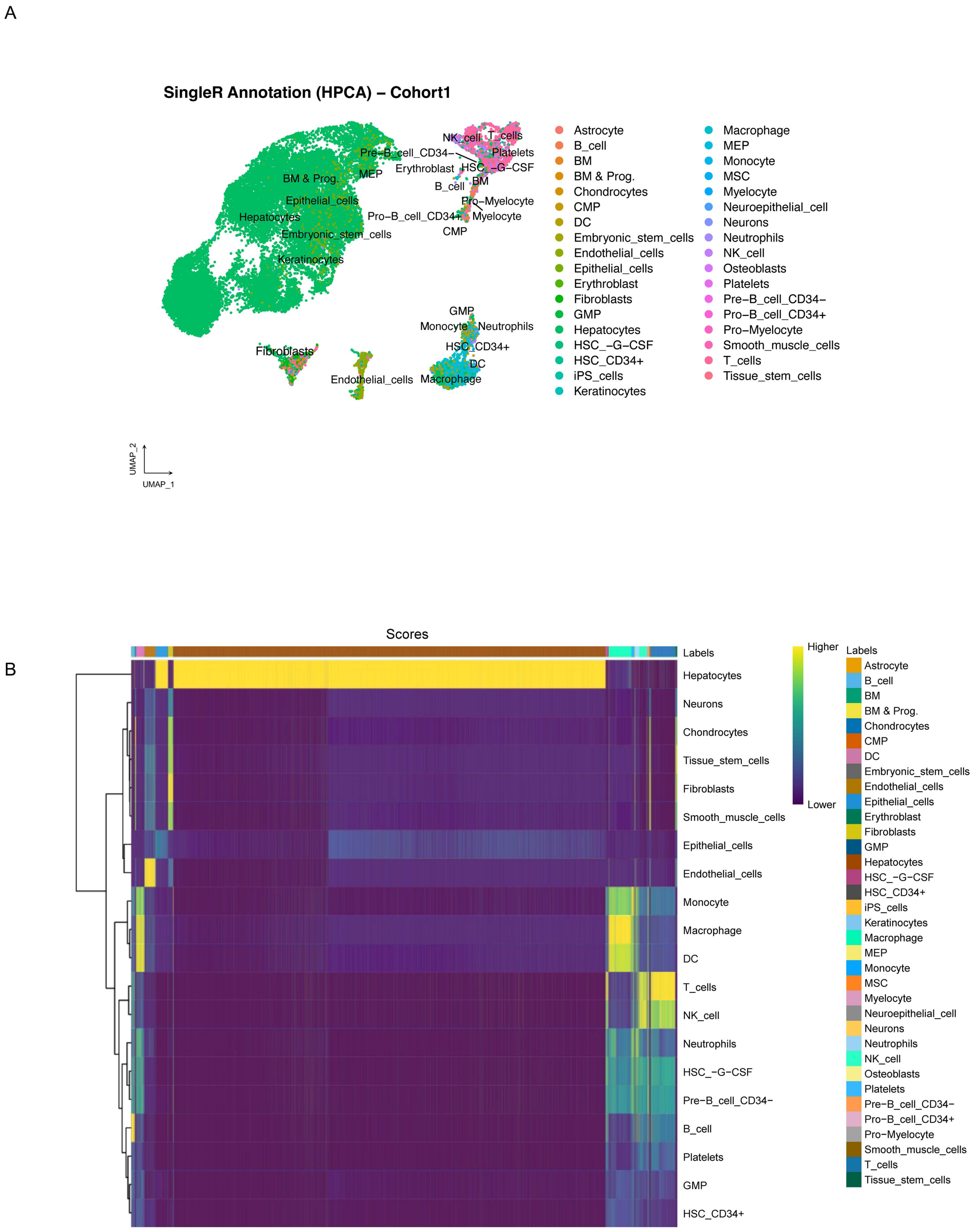

Supplementary Figure. 6 SingleR-based cell type annotation and reference score validation of the integrated scRNA-seq data.

(A) UMAP of SingleR-assigned cell type labels. UMAP embedding (Harmony-corrected) of cells colored by cell type labels assigned by SingleR (v2.8.0) using the Human Primary Cell Atlas (HPCA) as reference. The dominant cluster corresponds to Hepatocytes, consistent with the liver tissue origin of the samples. Smaller distinct clusters on the left contain immune cell populations including T cells, NK cells, B cells, Monocytes, Macrophages, and Dendritic cells, while the bottom cluster is enriched for Endothelial cells. These SingleR-derived labels were used as one line of evidence to guide manual cell type annotation.

(B) Heatmap of SingleR reference scores. Heatmap displaying the per-cell SingleR correlation scores against all reference cell types in the HPCA database. Columns represent individual cells grouped by their final SingleR-assigned label (indicated by the color bar at the top), and rows represent reference cell types. Color intensity indicates the score magnitude (yellow: higher similarity; purple: lower similarity). Diagonal enrichment — high scores concentrated along the assigned label row — demonstrates that the classification is specific and that each cell population is transcriptionally distinct from other reference categories.

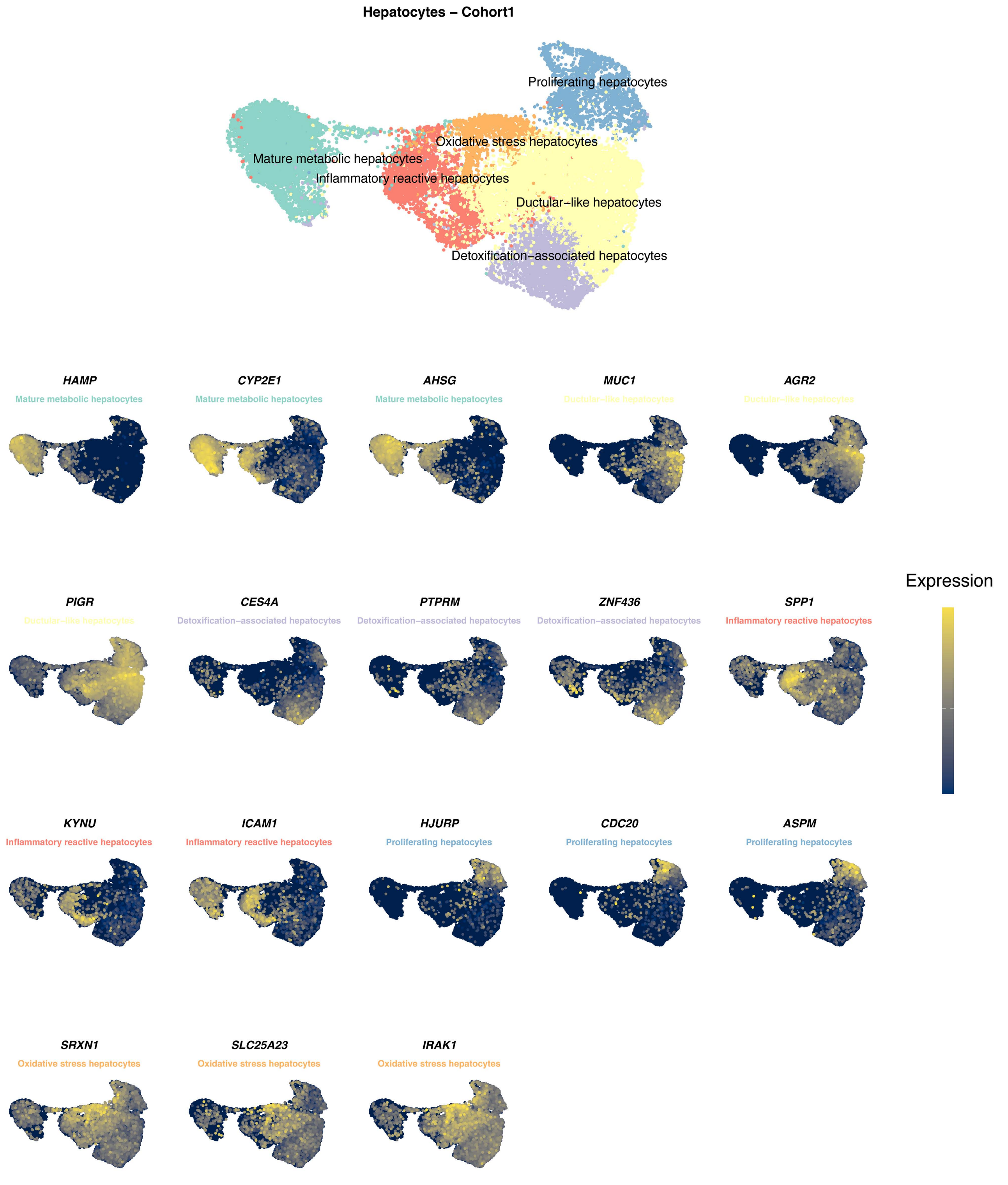

Supplementary Figure. 7 Hepatocyte subcluster annotation and marker gene expression before and after treatment.

UMAP of hepatocyte subclusters: mature metabolic, ductular-like, detoxification-associated, inflammatory reactive, proliferating, and oxidative stress hepatocytes. Feature plots showing expression of subcluster marker genes. Color scale indicates normalized log-transformed expression (dark blue: low; yellow: high). Color scales are shown individually for each gene and should not be compared directly across markers. Mature metabolic hepatocytes: HAMP, CYP2E1, AHSG; ductular-like hepatocytes: MUC1, AGR2, PIGR; detoxification-associated hepatocytes: CES4A, PTPRM, ZNF436; inflammatory reactive hepatocytes: SPP1, KYNU, ICAM1; proliferating hepatocytes: HJURP, CDC20, ASPM; oxidative stress hepatocytes: SRXN1, SLC25A23, IRAK1.
